# SERS detection of Biomolecules at Physiological pH via aggregation of Gold Nanorods mediated by Optical Forces and Plasmonic Heating

**DOI:** 10.1038/srep26952

**Published:** 2016-06-01

**Authors:** Barbara Fazio, Cristiano D’Andrea, Antonino Foti, Elena Messina, Alessia Irrera, Maria Grazia Donato, Valentina Villari, Norberto Micali, Onofrio M. Maragò, Pietro G. Gucciardi

**Affiliations:** 1 CNR-IPCF, Istituto per i Processi Chimico-Fisici, Viale F. Stagno D’Alcontres 37, I-98156, Messina, Italy

## Abstract

Strategies for in-liquid molecular detection via Surface Enhanced Raman Scattering (SERS) are currently based on chemically-driven aggregation or optical trapping of metal nanoparticles in presence of the target molecules. Such strategies allow the formation of SERS-active clusters that efficiently embed the molecule at the “hot spots” of the nanoparticles and enhance its Raman scattering by orders of magnitude. Here we report on a novel scheme that exploits the radiation pressure to locally push gold nanorods and induce their aggregation in buffered solutions of biomolecules, achieving biomolecular SERS detection at almost neutral pH. The sensor is applied to detect non-resonant amino acids and proteins, namely Phenylalanine (Phe), Bovine Serum Albumin (BSA) and Lysozyme (Lys), reaching detection limits in the μg/mL range. Being a chemical free and contactless technique, our methodology is easy to implement, fast to operate, needs small sample volumes and has potential for integration in microfluidic circuits for biomarkers detection.

Plasmonics has brought new revolutionary methods in the field of biomolecular detection^1^,^2^,^3^,^4^,^5^. Surface-enhanced Raman scattering^6^ (SERS) exploits the giant electromagnetic field enhancement (10^4^–10^8^) provided by localized surface plasmon resonances (LSPR) in metal nanoparticles (NPs)^7^, allowing one to tailor the molecular sensitivity to the atto-molar^8^ range and reach single molecule sensitivity, in special cases^9^,^10^. SERS has shown enormous application potentials in label-free detection of biomolecules^11^,^12^,^13^,^14^,^15^ and proteins^16^,^17^,^18^, adding to standard surface plasmon resonance sensors the spectral information, useful to get insights on the functional state of the biomolecules^19^. Different concepts of SERS-based biosensors have been demonstrated so far. Raman dye-labeled sensors exploit SERS-active labels (NPs coated with high Raman cross-section dyes and functionalized with antibodies against the target molecule) to spot proteins, permitting their indirect detection (the signal of the dye is monitored) also *in-vivo*^20^,^21^,^22^,^23^. Direct, label-free SERS sensors, are, however, desirable due to operational rapidity, simplicity and richness of information content (here the enhanced spectrum of the target molecule is acquired)^18^,^24^, since the Raman fingerprint of proteins, in principle, gives insight on their conformation and structure^19^,^25^. Label-free SERS detection of proteins in liquid environment has turned out to be a challenge, due to the difficulty to efficiently induce SERS-active aggregates in a solution containing biomolecules without altering their functionalities. The addition of NPs aggregates to protein solutions paved the way to quantitative SERS of uric acid in human serum with limits of detection (LOD) ~240 μM (equivalent to 40 μg/mL)^26^. An effective strategy to improve the sensitivity is to induce the NPs aggregation in presence of the target protein, e.g. via addition of acidified sulfate^27^. This yields SERS-active colloid-protein complexes in which the biomolecule is located at the NPs interstices (hot spots) allowing for detection of non-resonant proteins at concentrations down to 5 μg/mL (Lysozyme, Lys)^27^. The same concept has been tailored to optical fiber sensors that, taking advantage of sandwich NP-protein-NP structures, can push the sensitivity down to 0.2 μg/mL^28^. In both cases, however, the need of an external chemical agent to induce aggregation and the acidic environment required (pH 3) yield protein denaturation and hinder *in-vivo* applications. Other chemical/physical approaches to create efficient hot spots for SERS detection of biomolecules in liquid exploit hydrophobic interactions (LOD ~ 5 μg/mL for cytochrome C and Lys)^29^, heat-induced self-assembly (LOD ~ 50 nM for glutathione)^30^, aggregation of NPs with biocompatible coatings (LOD ~ 50 nM for cytochrome C)^31^, iodide-modified Ag NPs (LOD ~ 3 μg/mL for Lys, 300 μg/mL for BSA)^32^, mechanical aggregation of Au NPs in a microfluidic channel (LOD ~ 0.1 nM for BSA)^33^, state translation of nanoparticles from the wet to the dry state driven by evaporation (LOD of 1 pM for Cresyl Violet)^34^.

Optical forces^35^,^36^ can play an important role in this context, enabling the formation of efficient SERS hot spots in a controlled, contactless way^37^,^38^,^39^. Light exerts forces and torques on metal NPs, enhanced by the plasmon resonances^40^,^41^,^42^,^43^. When the energy of the laser field is far-off the LSPR, optical forces are dominated by the gradient force^36^,^39^,^41^ and can either attract^44^,^45^,^46^,^47^ or repel^43^,^48^ metal NPs from high field intensity regions, permitting to either trap metal NPs in the spot of a tightly focused Gaussian beam or push them in the hollow core of a Laguerre-Gauss beam. Instead, when the light is nearly-resonant with the particle LSPR, optical forces are dominated by radiation pressure^35^ and can be used to push metal NPs along the beam optical axis onto a substrate^49^,^50^,^51^,^52^. Pioneering experiments have shown that trapping forces permit to bring together individual metal NPs and create SERS-active dimers^53^. Optical tweezing of metal colloids allows the formation of SERS-active aggregates in liquid^54^ or inside lab-on-chip architectures^55^ to perform SERS detection of the organic compounds present in solution (pseudoisocyanine at 10 fM, naphtalenethiol at 50 μM). Metalized silica beads can be efficiently trapped and used for SERS detection of emodin, a purgative resin, at μM concentrations^56^. Optical trapping of NPs can even be performed with a photonic crystal cavity for controlled SERS detection of 4-aminothiophenol molecules in solution down to concentrations of 10 nM^57^. It is also possible to trap gold colloids aggregated in presence of BSA and detect the enhanced Raman scattering of the protein^58^. Illuminating with a laser beam Ag ions dispersed in a solution containing dye molecules it is possible the locally grow SERS-active Ag NPs and detect the presence of the dye molecules at the NPs hot spots^59^,^60^. The concept can be also implemented on a lab-on-chip platform^61^. Dynamic assembly of metal NPs by the plasmonic field generated in a metal film allowed to even reach single molecule SERS sensitivity^62^. Optical forces offer key advantages over the chemical/physical aggregation methods in terms of control of the process, contactless and chemicals-free operation, simplicity of operation, possibility of *in-vivo* applications. The potential of optically induced aggregation in the field of biomolecular SERS detection, however, has not yet fully demonstrated. In addition, the experimental configurations developed so far are based on the concept of aggregation via optical-trapping through the gradient force, i.e., exploiting the conservative part of the optical force. The other side of the coin, i.e. the possibility to exploit the radiation pressure to selectively push and aggregate metal NPs for SERS detection, remains largely unexplored. This latter approach represents a step forward in the development of SERS-based molecular sensors in liquid, since it allows one to use lasers with a broader wavelength range (no more limited by the LSPR of the NPs), it enables the controlled local spotting of metal nanoparticles on surfaces or even into living cells^52^ for local SERS analysis. Here we report on the implementation of a label-free, all-optical SERS sensor for biomolecular detection in liquid (in LIQUId SERS sensOR, hereinafter LIQUISOR) that exploits the radiation pressure to push gold nanorods on a surface and form SERS-active aggregates in buffered solutions of amino acids and proteins. We apply this methodology to detect Phenylalanine (Phe), Bovine Serum Albumin (BSA) and Lysozyme (Lys) at concentrations down to few μg/mL (50 nM for BSA, 100 nM for Lys). The LIQUISOR extends the concept of optical aggregation by laser trapping of metal NPs, is easy to implement, fast to operate, and has potential for integration in microfluidic circuits.

## Results and discussion

**Figure 1:**
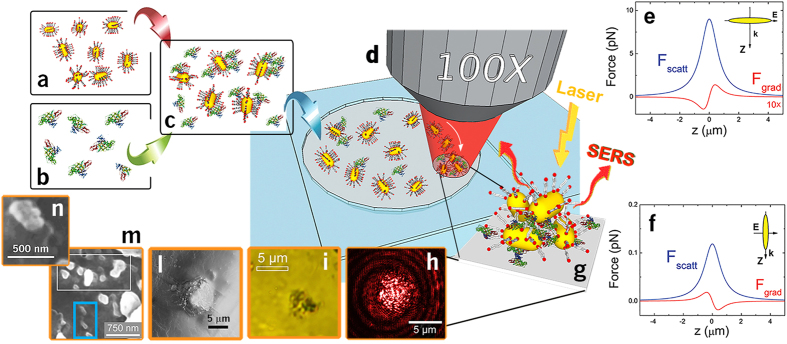
LIQUISOR concept. (**a**) The LIQUISOR employs gold NRs (35 nm diameter, 90 nm length), capped with Cetyltrimethylammonium Bromide (CTAB) and dispersed in deionized water. NRs are mixed with the target biomolecules dissolved in phosphate saline (**b**). Upon mixing, the biomolecules + PBS action destabilizes the CTAB and bind to the NRs (**c**). A tiny aliquot of the biomolecule-NRs solution (75 μL) is pipetted into a hemispheric glass microcell (**d**), sealed with a coverslip and put under a Raman micro-spectrometer. A laser beam is focused on a micron scale spot in liquid, close to the sidewalls of the microcell. Calculations of the optical forces show that for field polarization parallel to the NRs long axis, the gradient force (**e**, red line) has a repulsive nature, due to the blue shift of the laser energy with respect to the long axis LSPR. The scattering force (**e**, blue line) is even more intense. When the NR is oriented along to the propagation direction, *k*, i.e. field parallel to the short axis, the gradient force (**f**, red line) shows an attractive, “trapping” character, but also in this case the magnitude scattering force is markedly higher (**f**, blue line). In both orientations the net optical force acting on the NRs is positive, i.e., tends to expel the rod from the laser focus along the propagation direction. The relative *z* = 0 position refers to the center of the laser spot. The *z* axis is parallel to the wavevector *k* that points “downward” in our experimental configuration. The NRs are, consequently pushed towards the bottom of the cell, inducing the formation of SERS-active aggregates that embed the biomolecules (**g**). Laser scattering (**h**) and bright field images (**i**) show that aggregates can reach micron scale dimensions after several tens of minutes. (**l**) SEM image of a further larger aggregate. The zoom (**m**) highlights the presence NRs aggregated (white box) and kneaded with the biomolecules. Sparse individual NRs (blue box) are also visible on some points. (**n**) Locally NRs aligned in a side-by-side configuration can be found.

The working principle of the LIQUISOR is illustrated in Fig. 1 (see Methods for details). Gold nanorods (Fig. 1a) are added to a solution of biomolecules dissolved in phosphate buffered saline (PBS, Fig. 1b). The mixture (Fig. 1c) is pipetted in a glass microcell and placed under a Raman micro-spectrometer (Fig. 1d). The volume ratio is kept to 1:7 v/v, small enough to preserve the neutral pH (7.2) of the biomolecules solutions. Upon mixing, the biomolecules bind to the gold NRs^63^, due to the interplay between the electrostatic interaction with the positively charged cetyltrimethylammonium bromide (CTAB) bilayer of molecules surrounding the NRs, destabilization of the CTAB bilayer at physiological pH^64^ induced by the PBS and intercalation of the amino acid residues of the protein^5^,^65^,^66^,^67^. This yields the formation of biomolecule-NRs complexes (BIO-NRCs)^68^ in which individual NRs are stabilized by the protein layer in the solution. For BSA at room temperature, BIO-NRCs have a mean hydrodynamic radius (MHR) almost double with respect to the pristine NRs, as observed by dynamic light scattering (Supplementary Note 1 and Supplementary Fig. S1a). The MHR does not vary with time, indicating that the dimensions of the NRs, after a fast uptake of BSA from the solution, are stabilized. Extinction spectra confirm this result (Supplementary Fig. S2a), showing that even after 50 min from the NRs-protein mixing, the signal is still dominated by the LSPR fingerprint of individual NRs, characterized by a short axis resonance at 527 nm and a long axis resonance at 687 nm. Only a slight red-shift (1–1.5 nm) and broadening (20 nm) with respect to the original NRs is observed, that can be attributed to the change of local dielectric constant. These results allow us to conclude that the BIO-NRCs in solution are by far composed of individual NRs surrounded by some protein layer. The presence of NRs clusters (dimers or trimers), although cannot be excluded a-priori, has not been detected. Operation of the LIQUISOR is carried out by focusing the laser spot inside the microcell, near the side wall, in proximity of the bottom surface. To foster the aggregation, in fact, the BIO-NRCs must be conveyed optically in a region of few tens of microns. This is achieved by positioning the laser spot as close as possible to the rim of the hemispherical microcell (Supplementary Fig. S3), in the limited free space between the microcell sidewalls and the coverslip so to focus the laser in proximity of the cell bottom surface. We do not observe aggregation or SERS signal when the laser spot is focused into the solution, i.e. far from the side walls, as would be expected if SERS-active small clusters of protein-NRs would be spontaneously formed in the solution. We use a laser wavelength (632.8 nm) blue shifted with respect to the major axis LSPR of the rods at 687 nm (Supplementary Fig. S2b). For particles smaller than the laser wavelength, such as the NRs used in our experiments, the radiation force exerted by a Gaussian beam has two contributions^39^,^48^: a *gradient force* proportional to the gradient of light intensity, and a *scattering force* proportional to the light intensity and extinction cross section. The first component is conservative, it controls the operation of optical tweezers^36^,^39^, and it is generally increased by exploiting high numerical aperture objectives to create high intensity gradients. Instead, the scattering force is non-conservative and it is responsible for pushing particles along the light propagation direction^51^. We have the optical force components exerted by the laser field on a single NR (see Methods). Calculations are done for the two main configurations in which the field is polarized along the NR long and short axis (Fig. 1e,f, respectively) as a function of the rod position with respect to laser focus center (*z* = 0)^43^. For our experimental conditions the scattering force components (blue lines) always prevalent, by at least one order of magnitude, with respect to the gradient forces (red lines), no matter how the nanorod is oriented. The net force balance acting on the rod is, therefore, always positive, i.e. directed along the propagation direction *k*. This leads to the pushing of the nanoparticles along the optical axis towards the bottom of the cell, as experimentally observed (Supplementary Movie 1). Such process enables the dynamic accumulation of the BIO-NRCs present in solution onto the bottom surface of the microcell, in a zone around the laser focus, where they stick and aggregate, forming structures that can reach the size of several microns. Figure 1h,i show, respectively, the laser scattering image and the bright field image of two optically induced aggregates. Supplementary Fig. S4a displays the time series image of an optically induced aggregate of gold NRs in BSA. Between 0 and 20 min we observe a steep increase of the number of particles in the illuminated spot leading to an enlargement of the aggregate size. After 30 min the aggregate size saturates and reaches a steady state. The aggregate growth kinetics is shown in Supplementary Fig. S4b. Figure 1l shows the SEM image of a large aggregate produced after prolonged irradiation. The respective bright field images, before and after the aggregate formation, are in displayed Supplementary Fig. S5(a,b). A zoom of the SEM image (Fig. 1m) provides information on the BIO-NRCs organization. Sparse rods (cyan box) are visible having lengths of 150–200 nm and widths 80–100 nm, probably composed by individual rods surrounded by some protein layers. Most of the structures, however, show up with a more complex morphology, featuring dimensions in the 250–350 nm range (white box in Fig. 1m and Supplementary Fig. S5d). These structures are likely composed by few rods, surrounded and linked together by the protein. No preferential alignment along the laser field is observed. Some complexes (Fig. 1n) show rods aligned in a side-by-side configuration. In larger complexes, the BIO-NRCs seem to aggregate in a randomly oriented fashion. The extinction spectrum of the aggregate (Supplementary Fig. S2b, brown line) shows a broadening and red shift of the plasmon resonance^41^, that is what we expect when the NRs are near-field coupled. Such coupling makes them optically resonant at both the laser and the Raman wavelengths and SERS active^69^. As a consequence, the biomolecules embedded among the NRs experience an enhanced laser field and a strong re-radiation effect, leading to high SERS amplification^70^. In addition to optical forces, laser-induced heating of the NRs^71^ (the laser is quasi-resonant with the LSPR) may contribute in further increasing the protein uptake, thus enlarging the dimensions of the optically induced aggregate. An increased protein uptake is observed, in fact, in BSA mixed with NRs when the temperature of the solution is brought to 60 °C (Supplementary Fig. S1c). This is a consequence of the fact that BSA, alone, does aggregate into oligomers with increasing temperature (Supplementary Fig. S1b). This effect, combined with the thermally-induced structural rearrangement of NRs micellar capping^72^, may foster the re-organization of the individual BIO-NRCs the into larger SERS-active aggregates interlinked by the biomolecules, when optically confined at the bottom of the microcell.

**Figure 2:**
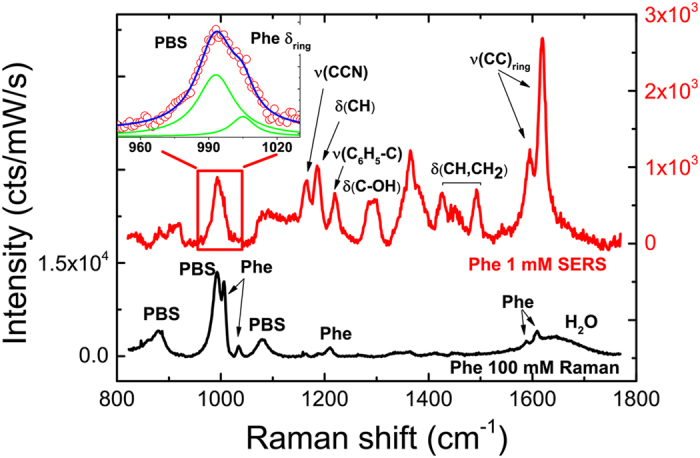
LIQUISOR detection of Phenylalanine. SERS spectrum of Phe 1 mM in PBS (red line) compared with solution phase Raman of Phe 100 mM in PBS (black line). Both spectra are taken in the same experimental conditions (wavelength 632.8 nm, objective 100X, power 6.7 mW), but for the integration times (10 s for SERS, 300 s for Raman). The spectra have been normalized to power and integration times, so the intensities can be directly compared. The Raman spectrum shows the most intense peaks of Phe at 1006, 1034, 1210, 1588, 1609 cm^−1^ (see Supplementary Table S1 for modes assignment) just emerging from to the stronger vibrational fingerprints of PBS (modes at 880, 993, 1080 cm^−1^), and of water (band at 1620 cm^−1^). The highest SERS enhancement is experienced by the vibrational modes in the ranges around 1200, 1400–1500 and 1600 cm^−1^ (see Supplementary Fig. S7). The inset is a zoom of the SERS (circles) in the 950–1030 cm^−1^ range and the relative fit (blue line), highlighting the Phe ring breathing mode at 1005 cm^−1^ emerging from the PBS band (here fitted by two Lorentzian line shapes, green lines). The SERS peaks are superimposed to a continuum background that has been subtracted for clarity.

Proof of principle operation of the LIQUISOR is provided by detecting L-Phe in PBS at concentrations of 1 mM (Fig. 2, red line), i.e. two orders of magnitude smaller than the limit of detection of normal Raman spectroscopy that is 100 mM (Fig. 2, black line and Supplementary Fig. S6)^73^. Phe is an aromatic amino-acid and an essential constituent of proteins, also involved in signaling functions and phenylketonuria pathologies^74^. A clear SERS fingerprint of the amino acid, markedly similar to its Raman counterpart and in agreement with what found by other groups^75^,^76^,^77^ is observed on an early stage aggregate. The most intense SERS vibrations (Supplementary Fig. S7 and Supplementary Table S1) are found at 1595 and 1616 cm^−1^ (in-plane ring stretching), in the 1400–1500 cm^−1^ range (C-H bending and scissors), at 1290 cm^−1^ (CH_2_), in the 1160–1220 cm^−1^ interval (C-CN stretch, CH bend, phenyl-C stretch). The typical ring breathing mode centered at 1005 cm^−1^ is detectable as emerging from the PBS band after fitting (inset of Fig. 2).

In order to evaluate the signal amplification provided by the LIQUISOR with respect to normal Raman spectroscopy, we introduce here the SERS gain, *G* (see Methods for definition and differences with respect to the SERS enhancement factor)^78^. *G* is calculated as the ratio between the SERS and the Raman signal intensities, normalized to power, integration time and concentrations of the target molecule. *G * is used here to provide a rapid quantitative estimate of the advantage in terms of signal amplification of the LIQUISOR method with respect to normal Raman spectroscopy. For Phe, considering the in-plane ring stretching at 1616 cm^−1^, we find that early stage aggregates can provide SERS gains *G *~ 6 × 10^3^.

**Figure 3:**
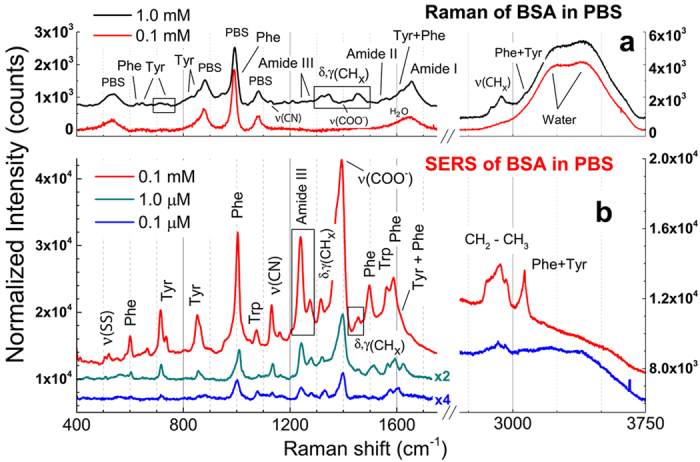
LIQUISOR detection of Bovine Serum Albumin. (**a**) Solution phase Raman spectra of BSA in PBS at 0.1 mM (red line) and 1 mM (black line). Spectra are shifted for clarity. At 0.1 mM the signal is dominated by the PBS and water vibrations (537, 880, 993, 1081 and 1640, 2800–3700 cm^−1^). At 1.0 mM the fingerprint of the BSA shows up both in the low energy (400–1700 cm^−1^) and the high energy (2700–3200 cm^−1^) range. The arrows indicate the BSA modes assignment carried out by comparison with Raman of BSA in powder state (Supplementary Fig. S8) and to the literature (see Supplementary Table S2). The Phe peak at 1006 cm^−1^ is visible only after fitting (inset of Supplementary Fig. S8). Integration time 30 s. (**b**) Upon formation of the aggregates a strong SERS signal is measured at concentrations of 0.1 mM (red), down to 1 μM (green) and 0.1 μM (blue). Integration times: 5 s at 0.1 mM, 10 s at 1 μM, 20 s at 0.1 μM. Spectra are offset for clarity. All the SERS and solution phase Raman spectra are rescaled to a common integration time of 30 s, so that the intensities can be directly compared. The left hand side scales refer to the low frequency modes. The right hand scales refer to the high frequency modes. The SERS peaks are superimposed to a continuum adsorbate-induced SERS background which at the lowest BSA concentration contains a component due to the water OH stretching in the high frequency part. Experiments are carried out with laser wavelength 632.8 nm, laser power 6.7 mW, microscope objective 100X.

**Figure 4:**
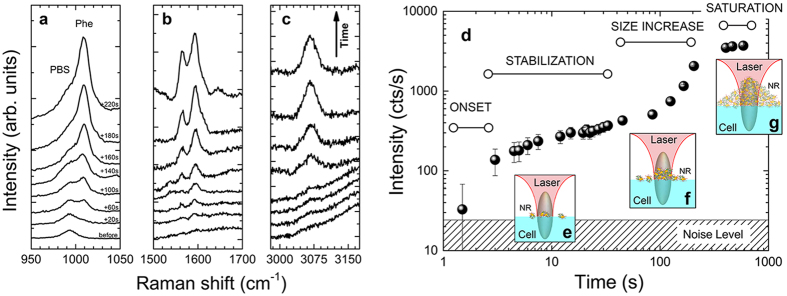
Dynamics of the SERS signal during the aggregate formation. SERS spectra of BSA are acquired at increasing times, as indicated by the row, (**a**) in the 950–1050 cm^−1^ (Phe ring breathing), (**b**) in the 1500–1650 cm^−1^ (aromatic amino acids and Amide I), and (**c**) in the 3000–3150 cm^−1^ (aromatic amino acids CH stretching) regions. The integration time for each spectrum is 20 s. The Amide I, shifted towards 1640 cm^−1^, suggests a β-sheets conformation of the protein in the hot spots. (**d**) SERS intensity of the phenylalanine ring breathing mode as a function of time during the BIO-NRCs aggregation. During the early stage aggregation, spectra are taken repeatedly with 1 s integration time. After 1 min, spectra are taken with 30 s integration. The intensities are normalized to the integration time. A continuous SERS signal increase is observed, even in the early stage aggregation phase. Error bars are related to the noise on each spectrum. The three insets (**e**–**g**) depict the NRs aggregation dynamics during the different time scales. The elliptical regions indicate the laser spot, namely its PSF, which is focused on the bottom of the liquid cell.

**Figure 5:**
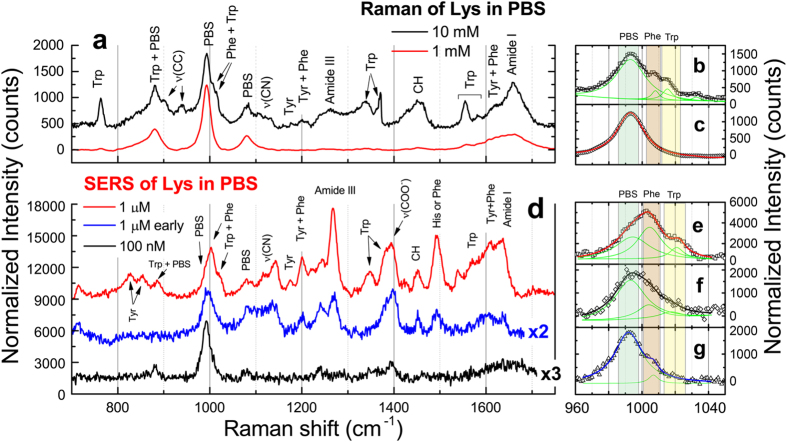
LIQUISOR detection of Lysozyme. (**a**) Solution phase Raman spectrum of Lys in PBS at concentrations of 1 mM (red line) and 10 mM (black line). Integration times are 30 s (10 mM) and 300 s (1 mM). The two spectra have been shifted for clarity and normalized to the integration time, so the intensities are directly comparable. The zoom in the 1000 cm^−1^ region of the 10 mM spectrum (**b**) highlights the presence of the Phe-Trp doublet peaked at 1008 cm^−1^ and 1015 cm^−1^, together with the intense PBS signal at 983 cm^−1^ Only the PBS contribution is visible at 1 mM spectrum (**c**). SERS spectra of Lys at concentrations of 100 nM (**d**, blue line and zoom in **e**) and 1 μM acquired on an early stage aggregate, after few minutes irradiation (**d**, black line and zoom in **f**), and after 60 min irradiation (**d**, red line and zoom in **g**). A tentative modes assignment (details in Supplementary Table S4) is carried out based on the Raman modes measured on the powder and in liquid at 10 mM (Supplementary Fig. S11). The SERS spectra were acquired with integration times of 60 s (1 μM early stage and 100 nM) and 40 s (1 μM). The spectra shown in the figures are rescaled to the same integration time, so to be directly comparable, and offset for clarity. In the zooms displayed in (**b**,**c**,**e**–**g**) the symbols refer to the experimental data which are fitted with a multi-peaks Lorentian model (black, red and blue lines) to extract the position of the single peaks of PBS, Phe and Tyr (green lines). Experiments are carried out with laser wavelength 632.8 nm, laser power 6.7 mW, microscope objective 100X.

The capabilities of the LIQUISOR to detect proteins have been evaluated on BSA, a model system composed by 607 amino acid units with a molecular weight of 66.5 kDa^79^. The LOD of BSA in PBS by normal Raman spectroscopy is ~1 mM (Fig. 3a, black line). At this concentration the most prominent vibrations of the aromatic amino acids (Phe and Tyrosine, Tyr), of the amide bands (I, II, III) and of the CH_x_ deformations, emerge from the PBS signal in the 600–1700 cm^−1^ range^80^. Similarly the CH stretching modes come out from the water OH vibrations in the high frequency region 2800–3100 cm^−1^ (see Supplementary Fig. S8 and Supplementary Table S2 for modes assignment). Detection of BSA in PBS by LIQUISOR is accomplished at decreasing concentrations, starting from 0.1 mM (Fig. 3b, red line), where no BSA can be detected by normal liquid-phase Raman (Fig. 3a, red line), down to 1 μM (Fig. 3b, green line) and 100 nM, where still a very strong signal is measured (Fig. 3b, blue line). The SERS spectra show very intense peaks in the same spectral ranges of the BSA vibrations (400–1700 cm^−1^ and 2850–3100 cm^−1^) found in solution-phase and in powder state (Supplementary Fig. S8)^75^,^77^,^81^, although different intensity ratios among the peaks are found. The spectra are well reproducible at all concentrations. The intensities from one aggregate to another (same concentration) can vary up to 50% (standard deviation), which is good enough to detect and distinguish proteins at different concentrations from 10^−7^ to 10^−4^ M. We can tentatively assign the vibrational modes comparing the SERS signal with the Raman spectra of solution-phase and powder BSA (Supplementary Table S2 and Fig. S8)^80^ and with the Raman spectra of the side-chain aromatic amino acids (Phe, Tyr, Tryptophan, Trp) characterized by a high Raman cross-section (Supplementary Fig. S9)^25^,^75^,^77^,^82^. This allows us to associate the strongest SERS peaks to the aromatic residues in the protein structure to the disulfide bridges (500 cm^−1^ region), the CH deformations (1300, 1450 cm^−1^), the COO^−^ symmetric stretching (1395 cm^−1^), the Amide III (1239, 1274 cm^−1^), the Amide I (1650 cm^−1^), the CH stretching (2820–3000 cm^−1^, band). Such mode assignment must be taken with care, since spectral shifts and intensity changes could occur as a consequence of the interaction of the protein with the residual surfactant layer and the gold surface. Control experiments have been carried out on the pure buffer solution and on the buffer added with CTAB-coated NRs, precipitated and aggregated on the bottom of the cell (Supplementary Fig. S10). Notably, PBS and CTAB^83^ do not show any significant spectral feature in the regions at 1004 cm^−1^ (Phe) and 1500–1650 cm^−1^, excluding major contributions to the SERS signal of the biomolecules, even at 100 nM concentration. Taking into account the SERS peaks positions found in different experiments reported the literature (Supplementary Table S3), we can assume as marker bands of the BSA the strongly enhanced SERS peaks related to the Phe ring stretching at 1004 cm^−1^, to the Tyr doublet in the 850 cm^−1^ region, to the Amide III (1240, 1275 cm^−1^) and Amide I (1650 cm^−1^) bands, to the Phe + Tyr modes in the 1585–1620 cm^−1^ and to the Phe + Tyr mode at 3066 cm^−1^. Some considerations can also be drawn on the structure of the BSA in the hot spots. Proteins interact with hydrophilic surfaces (such as the polar CTAB covering the NRs surface, as in our case) via hydrogen bonding with the peptide units exposed. Such interaction can affect the α-helical and β-sheets arrangement, leading to partial or total modification of the secondary structure^84^. BSA has been reported to unfold upon binding with CTAB-coated and citrate-stabilized gold NPs^65^,^85^. Unfolding occurs at the nanoparticle surface and fosters the aggregation of the protein via hydrophobic patch assembly^65^. Our measurements confirm a picture in which the protein at the hot spot is aggregated and strongly interacting with the NRs surface, featuring a somehow altered secondary structure. The strong enhancement of the COO^−^ symmetric stretching (1395 cm^−1^) not H-bonded, in fact, suggests an exposure and intercalation of the proteins hydrophobic side chains into the CTAB in strong electrostatic interaction with the surfactant bi-layer (see also Supplementary Note 2)^66^,^67^,^86^. The dominant presence of the aromatic CH stretching modes (between 3000 and 3100 cm^−1^), overwhelming the water OH bands, indicates that the liquid water is excluded from the hot spot region, due to the formation of a hydrophobic regions where the BSA molecules lay. The weakly enhanced Amide I band at 1650 cm^−1^, shifted towards 1640 cm^−1^ in some cases (*vide infra*), together with the absence of a clear signal around 940 and 1340 cm^−1^ (spectral fingerprints of the α-helical structure^25^) and with the higher intensity of the 1239 cm^−1^ peak with respect to the 1274 cm^−1^ one in the Amide III region^87^,^88^, suggest a prevalent content of β-sheets in the BSA aggregates. From the ratio between the intensities of the Anti-Stokes and Stokes SERS emission, calculated using the most intense BSA peaks (see Methods), we estimate that saturated aggregates under prolonged laser irradiation can reach temperatures higher than 40 °C (Supplementary Fig. S11). Such a temperature increase could justify the formation of β-amyloid structures at the hot spots^65^. The presence of the C_β_-S-S-C_β_ disulfide bridges features in the 500–550 cm^−1^ region shows, however, that the protein has still some form of tertiary structure (Supplementary Note 4), suggesting only partial modification of the protein conformation, not as extensive as the structural changes occurring in protein fibrillation. In order to study the operation dynamics of the LIQUISOR we have acquired consecutive SERS spectra during the optical aggregation of BIO-NRCs. Figure 4a–c show that the enhanced BSA fingerprint emerges from the PBS background in the first few seconds from the laser irradiation and the signal keeps increasing during the following minutes up to a saturation level. The aggregation follows two different time scales (Fig. 4d). The onset of the aggregate formation is observed after few seconds from irradiation when first early stage aggregates are formed from interacting BIO-NRCs within the laser focal spot (Fig. 4e). The aggregates stick on the cell sidewall and stabilize in the next few tens of seconds (20–60 sec), producing a stronger SERS signal of the biomolecule. On longer time scales (1–10 min) the aggregate repeatedly increases its dimensions due to the capture of further BIO-NRCs, adding up proteins and hot spots sites (Fig. 4f), further enhancing the SERS signal. The process keeps going with time, producing aggregates that can reach the size of several microns, due to the fact that the optical forces push the BIO-NRCs all around the laser spot. The SERS signal, however, saturates typically after some tens of minutes (10–30 min). Saturation occurs when the BIO-NRCs have totally filled up the actual laser focal volume (Fig. 4g), whose dimension can be assumed of the order of the Point Spread Function (PSF)^89^. From this moment on the addition of further NRs, laying outside the focal spot, does not contribute to the detected SERS signal, due to the confocal arrangement of the detection system. To investigate the LOD of BSA we further diluted the protein to 50 nM and 10 nM. The spectrum at 50 nM (Supplementary Fig. S12) shows the same fingerprint observed at higher concentrations, with peaks well distinct with respect to PBS and CTAB. After saturation of the aggregate, the signal remains stable even at the smallest concentrations, suggesting that no significant thermal-induced lateral diffusion of the NRs occurs^90^. BSA at 10 nM, conversely, yields unstable BIO-NRCs complexes. The SERS signal, even upon prolonged laser irradiation, cannot be distinguished in an unambiguous way from the CTAB signal of precipitated NRs. Very likely, BSA at 10 nM is not sufficient to surround completely the NRs, stabilize them in the solution and provide the conditions necessary for the optically induced aggregation. We can therefore assert that the LOD of BSA in PBS through the LIQUISOR is between 10 nM (0.66 μg/mL) and 50 nM (3.3 μg/mL). A SERS gain *G *~ 10^5^ is found on saturated aggregates. Here *G* is calculated as the intensity ratio between the SERS signal of the Phe mode (1004 cm^−1^) at 100 nM and the respective Raman signal at 1 mM, after normalization to power, integration times and concentration (compare inset of Supplementary Fig. S10 with inset of Supplementary Fig. S8). *G* tells us that if we wait enough time to saturate the laser spot with interacting BIO-NRCs, the LIQUISOR provides a signal increase of 5 orders of magnitude with respect to a normal Raman spectrum. We can estimate also the SERS enhancement factor, *EF* (see Methods for definition) to obtain information on the amplification provided by the single nanostructure. To estimate the number of probed molecules in the SERS experiment, *N*_SERS_, we exploit SEM and DLS results (see Methods for details). DLS gives information on how many molecules, *N*_mol_, we have per nanostructure, SEM on how many nanostructures, *N*_nano_, we expect in the laser focus. *N*_SERS_ will be equal to 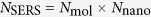. For BSA in PBS at 0.1 mM we estimate *N*_SERS_ ~ 10^4^ ÷ 10^5^ (see Methods). A ratio 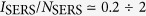 counts/molecule is therefore measured for the Phe peak at 1004 cm^−1^ (Fig. 3b, red line). For the solution-phase Raman measurement the number of probed molecules, *N*_Raman_, will be proportional to the molecular concentration in the solution, *c*_Raman_, and to the microscopic volume probed by the objective, calculated as the volume of the 3D PSF at 633 nm (see Methods for details)^89^. Our reference measurement has been carried out at a concentration of 1 mM where we estimate to have *N*_Raman_ ~ 10^6^ molecules, leading to a ratio 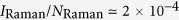 counts/molecule on the Phe peak at 1004 cm^−1^ (inset of Fig. S8). The enhancement factor provided by each nanostructure turns out to be between 10^3^ and 10^4^, in agreement with values found on near-field coupled NRs produced by electron lithography^91^. Indeed, this value refers to the assumption that all the molecules bound to the NRs are contributing to the SERS signal. The largest signal enhancement, instead, is expected from those molecules located at the hot spots. Such an overestimate of *N*_SERS_ let’s us to conclude that the *EF* value calculated above is a conservative, lower bound of the real enhancement factor. Geometrical considerations bring us to estimate that less than 10% of the molecules absorbed on the total NR area are located in the hot spot of a NRs dimer, when this is arranged in a tip-to-tip configuration (see Methods), yielding an estimated *EF* one order of magnitude larger. We finally demonstrate the operation capabilities of the LIQUISOR concept on Lysozyme (Lys). Lys is a enzyme featuring 129 peptide units and 14.4 kDa molecular weight, whose raised levels in plasma may be a useful biomarker of atherosclerotic cardiovascular disease and response to therapy^92^. Lysozyme does aggregate in presence of gold NPs at physiological pH^68^. Its SERS detection has been shown at concentrations down to 3–5 μg/mL in liquid environment^27^,^32^. The Raman LOD of Lys in PBS is 10 mM (Fig. 5a, black line). At this concentration the spectrum shows main vibrational contributions (Supplementary Table S4)^93^ due to the exposed aromatic amino acids (several strong peaks in the 700–1600 cm^−1^ range), to the CC stretching (900, 938 cm^−1^), to the CN stretching (~1100 cm^−1^), to the Amide III (1240, 1260 cm^−1^), to the CH deformations (band ~1450 cm^−1^) and to the Amide I (1658 cm^−1^). The doublet associated to Phe at 1008 cm^−1^ and Trp at 1015 cm^−1^ (more evident in the spectrum in powder state, Supplementary Fig. S13, black line) clearly shows up from the intense PBS scattering at 993 cm^−1^ (Fig. 5b) after fitting (see Fig. 5b). Such doublet can be assumed as a spectral marker distinguishing Lys from BSA. Decreasing the concentration to 1 mM (Fig. 5a, red line), only small perturbations to the PBS spectrum in correspondence of the Trp modes (700, 1300–1550 cm^−1^) are observed in the Raman spectrum. The Phe-Tyr doublet around 1000 cm^−1^ is totally covered by the PBS signal (Fig. 5c). Detection of Lys by LIQUISOR is achieved adding gold NRs to a Lys solution in PBS, in a 1:4 v/v ratio. Lys binds to the NRs because of hydrophobic interaction^94^ yielding the formation of BIO-NRCs. The mechanism is different from BSA. Here the BIO-NRCs complexes formation is mediated by the destabilization of the CTAB bilayer in PBS at physiological pH^64^ and the consequent hydrophobic interaction between the CTAB alkyl chain and the protein nonpolar residues. This mechanism overcomes the repulsive interaction between any residual CTAB molecules^95^ and the Lys, both positively charged (the isoelectric point of Lys is 11.3), yielding the formation of protein-based aggregates at physiological pH^68^. At concentrations of 1 μM SERS of Lys is visible typically after 2–5 min of irradiation (early stage aggregate in Fig. 5d, blue line). Enhanced vibrational fingerprints are observed around 1000 cm^−1^ (Fig. 5f), where the Phe-Trp doublet can be clearly discerned from the PBS signal, as well as in the 1100–1650 cm^−1^ range. More intense signal is obtained after 60 min irradiation (Fig. 5d, red line), when we have obtained a saturated aggregate. Again, spectra are found to be reproducible with intensity errors comparable to what found with BSA. The Phe-Trp doublet (Fig. 5e) now overwhelms the PBS scattering, with peaks slightly shifted and broadened, probably due to interaction with the CTAB environment. SERS is also observed in the CN stretching region (1100–1140 cm^−1^, Supplementary Table S4), in the Amide III region (1246, 1268 cm^−1^), in correspondence to the COO^−^ stretching (1395 cm^−1^) and of the Amide I region (shifted to 1637 cm^−1^), together with peaks from aromatic amino acids, mainly Trp, Tyr and Phe, in the 750–1630 cm^−1^ range (indicated in Fig. 5d and listed in Supplementary Table S4). We finally decrease the concentration to 100 nM (Fig. 5d, black line). In spite of the weak signals measured, a clear spectral fingerprint of Lys detection is found in correspondence of the Phe ring breathing at 1006 cm^−1^ (Fig. 5g) and the COO^−^ stretching at 1395 cm^−1^, zones in which neither PBS or CTAB provide important contributions. Subtraction of the PBS background allows us to better highlight the presence of the Lys vibrational peaks (Supplementary Fig. S14) in comparison with the signals from CTAB and PBS. We can therefore asses a LOD of 100 nM (1.4 μg/mL). We can compare here the SERS gains of a saturated and early stage aggregate, calculated as the ratios between the Phe mode intensities in the SERS spectra (1 μM, Fig. 5e,f) and the normal Raman spectrum (10 mM, Fig. 5b). For the saturated aggregate we find a value ~7 × 10^4^, comparable to what observed for BSA in the same saturated conditions. For the early stage aggregate we have a value ca. one order of magnitude smaller, related to the fact that the focal laser spot is not yet filled by the BIO-NRCs complexes, highlighting the importance of obtaining fully saturated aggregates to maximize the sensitivity of the LIQUISOR methodology.

## Conclusions

In conclusion, we have developed a novel methodology to perform SERS detection of biomolecules in buffer solution exploiting optical forces to induce a controlled aggregation of plasmonic nanorods. Detection of Phe, BSA and Lys is demonstrated at physiological pH, reaching sensitivities of few μg/mL, SERS gains ~10^5^ with respect to Raman and single nanostructure SERS enhancement factors up to 10^4^–10^5^. Our biosensor concept operates in liquid, the natural environment of biomolecules, is of rapid use (tens of seconds), experimentally simple (standard micro-spectrometers and commercial nanorods are used), reliable and intrinsically scalable to lab-on-chip devices. Our methodology permits to exploit laser sources that are nearly-resonant with the LSPR of the NPs and can, therefore, be complementary used besides standard techniques of optical aggregation via trapping in tightly focused beams, generally accomplished far detuned from the plasmonic response. Specificity can potentially be added to the LIQUISOR by using NPs functionalized with aptamers or antibodies capable to capture the target biomolecules in liquid, thus enabling specific detection of pathology biomarkers in liquid environment. Higher sensitivity can be potentially achieved using gold NRs adopting the spermine or iodide-modified approach^32^ to completely remove the surfactant layer, or using silver nanoplatelets^43^. The use of laser beams in the optical transparency window of biological tissues could enable the application of our scheme in combination with optical injection of nanoparticles into living cells^52^ for *in-vivo* SERS biomolecular detection.

## Methods

### Nanorods

Commercial gold nanorods (35 nm diameter, 90 nm length) are purchased from Nanopartz and used as received. They are dispersed in deionized (DI) water at a concentration of 0.05 mg/ml, equivalent to ca. 3 × 10^7^ rods/μL; the solution contains <0.1% ascorbic acid and <0.1% Cetyltrimethylammonium bromide (CTAB) surfactant preventing spontaneous re-aggregation. The solution varies between pH = 3–4. The capped rods have a positive ζ-potential (+40 mV).

### Proteins and proteins-NRs solutions

Bovine Serum Albumin, Lysozyme and Phenylalanine are purchased from Aldrich in lyophilized powder state. Protein buffered solutions at various concentrations are prepared by mixing the suitable amount of protein powder with a 200 mM of Phosphate Buffer Solution (pH 7.2). PBS is prepared by dissolving Na_2_HPO_4_ (14.94 g) and NaH_2_PO_4_ (5.06 g) in 200 mL of DI water. Following this procedure we prepared samples containing BSA at concentrations of 10^−3^–10^−8^ M, Lys at 10^−6^–10^−7^ M, Phe at 10^−3^ M. The NRs-proteins solutions are prepared by mixing the nanorods with the proteins dissolved in PBS in a volume ratio ranging from 1:7 v/v (Phe, BSA) and 1:4 v/v (Lys). All the solutions are prepared and used at room temperature.

### LIQUISOR setup and operation

The LIQUISOR operation is carried out with a LabRam HR800 Raman confocal Micro-Spectrometer (Horiba Jobin Yvon) coupled to a He-Ne laser (λ = 632.8 nm); the laser beam is focused by means of either a 50X (Olympus M-Plan, NA = 0.75, WD = 380 μm) or a 100X (Olympus M-Plan, NA = 0.90, WD = 210 μm) microscope objective mounted on a Olympus BX41-microscope working in a straight configuration. The laser power on the sample is 6.7 mW, enough to apply a sufficient radiation pressure on the nanorods for process activation. Optical aggregation is also possible with long working distance microscope objectives (Olympus LMPlanFl, NA 0.5, WD = 10.6 mm), provided we use higher laser power (13 mW). For these experiments an XploRA PLUS (Horiba) setup is used mounting a laser diode emitting at 638 nm. This latter system has been used for dynamic aggregate growth studies.

75 μL of the biomolecule-NRs solution is pipetted into a glass microcell consisting of microscope slides with hemispherical cavities (15–18 mm diameter, 0.5–0.8 mm depth) purchased from Marienfled GmbH (ref. 13 200 02). The microcells are covered with glass coverslips 170 μm thick purchased from Forlab. All the glasses are washed by immersion in a deionized watery solution (1% v/v) of HELLMANEX III detergent for 10–15 min, followed by rinsing in DI water in order to remove the residual detergent. Finally they are washed with ethanol and dried in air. The SERS signal of the BIO-NRCs is collected via the same illumination objective, in backscattering, dispersed by a 600 l/mm grating and detected through a Peltier-cooled silicon CCD (Synapse and Sincerity, Horiba Jobin Yvon). Spectra are typically acquired with integration times from seconds to tens of seconds.

### Scanning Electron Microscopy

SEM analyses were performed by a field emission Zeiss Supra 25 microscope. Prior to investigation the residual NR-protein solution is carefully extracted from the glass microcell and the sample is analyzed under vacuum by using an accelerating voltage of 2 KV, to avoid the damage of the sample.

### Optical forces calculations

The radiation force exerted by a Gaussian beam of power *P* and waist *w*_0_, propagating along the direction 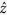, on a small scatterer (dipole approximation) immersed in a medium (water, *n*_m_ = 1.33) with refractive index *n*_m_ and permittivity 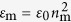 is given by^39^,^48^:


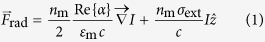


where 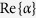 is the real part of the polarisability, 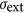 the extinction cross section, *c* is the speed of light, and 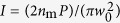 is the incident light intensity in the medium.

We estimate optical forces on our nanorods by modeling them as prolate spheroids with semi-axes *a*_1_* = *45 nm* > a*_2_* = a*_3_ = 17.5 nm that match the nanorods semi-dimensions. Thus, to calculate the polarisability we can use the Clausius-Mossotti relation as modified for ellipsoids^96^, and the optical constants measured for gold by Johnson and Christy^97^. The polarisability of a small spheroid illuminated along one of its principal axis is^96^:


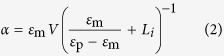


where ε_p_ is the spheroid (complex) permittivity, *V* the spheroid volume, and *L*_*i*_ are geometrical factors to be considered when the field is polarised along the principal axis *i* = 1, 2, 3. These geometrical factors are determined in terms of the particle eccentricity, 
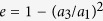
, and for a prolate ellipsoid are^96^:


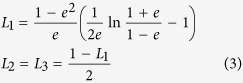


We considered both the cases in which the prolate spheroid has its long (*a*_1_) or short (*a*_2_ = *a*_3_) axis aligned with the field direction. From the particle polarisability we can easily obtain the extinction cross-section as:


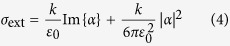


where 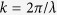 is the wave vector, 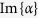 and 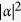 are the imaginary part and the square modulus of the spheroid polarisability, respectively.

### Temperature estimation

Estimating the temperature from the SERS data is a critical task. The temperature of the aggregate can be retrieved from the anti-Stokes/Stokes intensity ratio 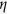, according to the relation^98^:


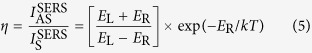


where 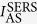 is the SERS anti-Stokes intensity, 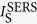 the SERS Stokes intensity, *E*_L_ is the laser photons energy, *E*_R_ the energy of the vibrational mode considered, *k* the Boltzmann constant and *T* the temperature (in Kelvin). Here we calculate *η* on all the BSA peaks (Supplementary Fig. S11, black symbols) and fit the data using Eq. 5 (fits are displayed in Supplementary Fig. S11, black line) to find out the sample temperature. A value of 316 K (43 °C) is found from the raw experimental data, i.e. without considering any wavelength dependence of the SERS enhancement factor^99^,^100^,^101^. Such a dependence is related to the re-radiation properties of the NPs that enhanced the Raman signal and is expected to alter the 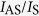 ratio, causing a higher enhancement of the Stokes scattering with respect to the anti-Stokes, with a consequent underestimate of the sample temperature (Supplementary Note 3). As a first approximation, we can account for such a re-radiation effect relating it to the extinction spectrum of the aggregate *Q*_e_(*λ),* i.e. rescaling the 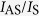 ratio of a quantity 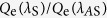 (Supplementary Note 3 and Supplementary Fig. S11, red symbols). The extinction spectrum is assumed to be independent from the temperature^90^. By fitting the re-scaled values (Supplementary Fig. S11, green line) we find an actually higher temperature T = 335 K (62 °C). The systematic upshift of the low energy data points with respect to the best fit curve, even after re-scaling, suggests that even higher temperatures (~80 °C) can be reached. The discrepancy between the temperatures calculated using the high and the low energy Raman modes could, however, be due to a steeper wavelength dependence of the re-radiation *EF*, even if not as steep as the one reported in ref. 99 (see Supplementary Note 3 for further discussion).

### SERS Gain and SERS Enhancement factors: definitions

To provide a estimate of the advantage of the LIQUISOR with respect to normal Raman spectroscopy we calculate two quantities, the SERS gain, *G*^78^, and the SERS enhancement factor, *EF*^7^. The SERS gain, *G*, is calculated as the ratio between the SERS (*I*_SERS_) and Raman (*I*_Raman_) intensity of a reference vibrational peak, normalized to the different powers (*P*_SERS, Raman_), integration times (*T*_SERS,Raman_) and molecular concentrations (*c*_SERS, Raman_) used in the experiment:


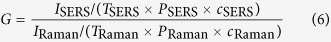


*G* provides, at a glance, quantitative information on the signal gain that one has to expect from a specific SERS sensor with respect to a reference Raman experiment (in our case, Raman spectroscopy in liquid), assuming that all the experimental parameters, such as objectives, laser wavelength, spectrometer etc. are the same.

On the other hand, we can define the SERS enhancement factor, *EF* 7, as the ratio between the SERS (*I*_SERS_) and Raman (*I*_Raman_) intensities normalized to the different powers (*P*_SERS,Raman_), integration times (*T*_SERS,Raman_) and number of probed molecules (*N*_SERS, Raman_)


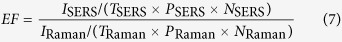


The *EF* is a measure of the signal amplification experienced by each molecule on each nanostructure, giving information on the field enhancement provided by the nanostructure itself. The *EF* is challenging to calculate since critical information on the number of probed molecules in the SERS experiment is required. Assumptions of single/few monolayers coverage, as well as estimates of the extension of the *hot spot* regions are needed in the case of SERS from nanostructured surfaces^70^, whereas assumptions on the total uptake of the molecules on the nanoparticles’ surface are required when performing measurements in liquid^101^. *G* has the advantage of being free from any overestimation error made when calculating the probed molecules ratio in the *EF. G* is, by definition, independent on the target molecule concentration, but depends on the aggregate size. *G* saturates to a constant value once the aggregate has filled the scattering volume probed by the microscope objective, since molecules outside such a volume can be considered “out of focus,” providing negligible contribution to the total optical signal.

### SERS Enhancement calculation: evaluation of the number of probed molecules

As shown by Eq. 7, the SERS enhancement factor 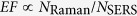, requires knowledge of *N*_SERS_ and *N*_Raman_. The number of probed molecules in the solution-phase Raman experiment can be calculated from


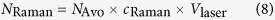


where *N*_Avo_ = 6.022 × 10^23^ is the Avogadro number, *c*_Raman_ is the molar concentration and *V*_laser_ the diffraction limited volume probed by the microscope objective. *V*_laser_ can be estimated calculating the volume of PSF of a TEM_00_ laser beam at wavelength λ, focused in air by an objective with numerical aperture *NA*. The PSF is well approximated by an prolate ellipsoid having semi-axes 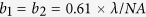 and 
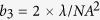
, where *b*_3_ is the semi-axis in the light propagation direction^89^. The volume of the focused laser spot, therefore, will be 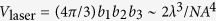. For λ = 633 nm and *NA* = 0.9, we find *V*_laser_ ~ 1.2 μm^3^. Consequently, the number of probed molecules will be 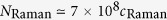, where *c*_Raman_ is expressed in mol/L (M).

The number of molecules probed in SERS can be estimated as the product 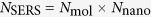 between the number of molecules surrounding each nanostructure, *N*_mol_, times the number of nanostructures present in the laser spot, *N*_nano_. To calculate of *N*_mol_ we use the information on the NRs’ hydrodynamic radius measured by DLS when they are mixed with BSA. The hydrodynamic model for a rod of length *L* and diameter *d*, predicts that the radius *R*_e_ of the sphere of equal hydrodynamic volume (that is what is measured by DLS) is given by 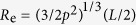 with 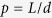
102. NRs in their native solution have a mean hydrodynamic radius *r*_NR_ = 35 nm (Fig. S1a, green symbols). This is in agreement with what expected for a 35 × 90 nm (diameter × length) cylindrical NR uniformly surrounded by a CTAB bilayer (~3 nm length)^103^ according to the hydrodynamic rod model^102^,^104^. Using the same model, we find that the increased NRs hydrodynamic radius, *r*_NR-BSA_ = 65 nm, measured upon addition of BSA in PBS (Fig. S1a) is compatible with the formation of a protein bilayer around the NRs. On the other hand, BSA in PBS at 0.1 mM (the condition in which DLS was carried out) shows a mean hydrodynamic radius of 6 nm, i.e. 2 times larger that what is expected for a single BSA molecule (ellipsoid with semi-axes *a*_1_ = *a*_2_ = 2 nm, *a*_3_ = 7 nm) according to Perrin’s model and experimentally measured, indicating the formation of some protein-protein complex^105^. We can use the information on the hydrodynamic volume increase, ΔV_NR_, due to the protein uptake by the NRs, to roughly estimate the average number of proteins captured. More specifically we have 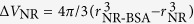 ~10^6^ nm^3^, whereas the hydrodynamic volume of the BSA is 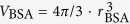
~ 10^3^ nm^3^. This yields an estimate of *N*_mol_ of the order of 10^3^.

The number of NRs in the laser focus, *N*_nano_, can be estimated in the ideal situation in which we consider a saturated aggregate where the NRs, surrounded by a protein bilayer, are closely packed and totally fill the focal laser spot semi-volume (the laser is focused slightly below the glass cell bottom, as sketched in Fig. 4g) *V*_laser_ ~ 1.2 μm^3^. Calling 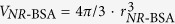 the hydrodynamic volume of the NR-BSA complex (V_NR-BSA_ ~ 10^−3^ μm^3^), *N*_nano_ can be roughly estimated as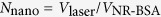, i.e. *N*_nano_ ~ 10^3^, yielding a number of probed molecules *N*_SERS_ ~ 10^6^. In the more realistic situation shown by the SEM pictures, however, we are more likely probing few tens of NRs dimers or trimers within our laser spot, yielding *N*_SERS_ ~ 10^4^–10^5^. This number refers to a situation in which all the molecules bound the nanostructure experience the same SERS enhancement. If we consider a NRs dimer arranged in a tip-to-tip configuration, however, we expect that the only molecules located at one edge of each nanorod will experience the SERS enhancement. For NRs in a side-by-side arrangement, a fraction of the molecules bound to half the lateral area of each rod (the side exposed to the nanocavity), will experience the SERS enhancement. For 35 × 90 nm nanorods, like ours, this amounts to less than 10% of the molecules absorbed on the total NRs surface, for a tip-tip configuration, and to less than 40% for the side-by-side arrangement.

## Additional Information

**How to cite this article**: Fazio, B. *et al*. SERS detection of Biomolecules at Physiological pH via aggregation of Gold Nanorods mediated by Optical Forces and Plasmonic Heating. *Sci. Rep.* **6**, 26952; doi: 10.1038/srep26952 (2016).

## Supplementary Material

Supplementary Information

Supplementary Movie S1
