# Supplementary material for: SERS detection of Biomolecules at Physiological pH via aggregation of Gold Nanorods mediated by Optical Forces and Plasmonic Heating
Source: Sci Rep. 2016 Jun 1;6:26952. doi: 10.1038/srep26952 (PMC4887892; doi:10.1038/srep26952)
Supplement: Supplementary Information [file srep26952-s1.pdf]

Supplementary Information

SERS detection of Biomolecules at Physiological pH via aggregation of Gold Nanorods mediated by Optical Forces and Plasmonic Heating.

Barbara Fazio,[1] Cristiano D'Andrea,[1,a] Antonino Foti,[1,b] Elena Messina,[1] Alessia Irrera,[1] Maria Grazia Donato,[1] Valentina Villari,[1] Norberto Micali,[1] Onofrio M. Maragò,[1] and Pietro G. Gucciardi[1,c,*]

[1]CNR-IPCF, Istituto per i Processi Chimico-Fisici, Viale F. Stagno D'Alcontres 37, I-98156, Messina, Italy

[a] Now at MATIS IMM - CNR, via S. Sofia 64, 95123 Catania, Italy

[b] Also at Dottorato di Ricerca in Fisica, Università di Messina, Viale F. Stagno D'Alcontres 31, 98166 Messina Italy

[c,*] Email: gucciardi@me.cnr.it

Supplementary Note 1

Dynamic light scattering experiments are carried out by using the Photon Correlation Spectroscopy technique. A He–Ne laser source (10 mW), polarized orthogonal to the scattering plane, is focussed onto the sample and the scattered light is collected at 90° by using a self-beating detection mode. In order to avoid the electric signal of the photomultiplier after pulse, two photomultipliers are used in a pseudo-cross correlation mode at the same scattering angle.[1] For collection of the polarized and depolarized scattered light, a Glan-Thomson analyser is placed in the scattered beam. The polarization analysis of the light scattered by the gold rods in solution (the depolarization ratio being about 30%) allows for separating the translation and rotation dynamics. During the experiment each sample is put in a glass cuvette positioned inside a thermostat in order to perform the experiment in a thermal equilibrium condition.

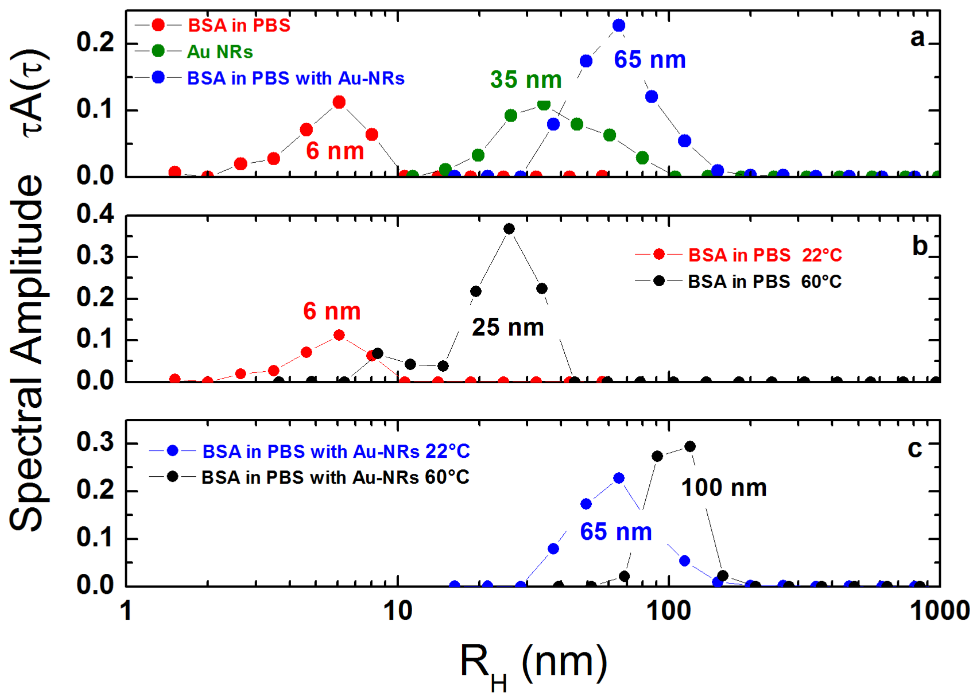

Figure S1: DLS measurements on NRs and BSA-NRs complexes. The translation diffusion coefficient of the NRs yields a mean hydrodynamic radius of about 35nm (a, green symbols). At room temperature BSA solution in PBS at 10^{-4} M shows a mean hydrodynamic radius of about 6 nm (a, red symbols) and the scattering is almost totally polarized due to the folded conformation at the investigated pH = 7.2. At T = 60°C there is an evident temperature-induced aggregation of BSA molecules, which gives rise to oligomers with hydrodynamic radius of about 25 nm (b, black symbols). This population increases with time if the solution is kept at 60 °C, likely as a result of partial protein conformational change. Upon adding gold rods in the BSA solution at room temperature the depolarization ratio becomes about 15% and the translation diffusion coefficient of the scattering particles gives a mean hydrodynamic radius of about 65 nm (c, blue symbols). The increase of the particles size suggests the existence of aggregates composed by gold rods surrounded by BSA molecules, likely stabilized by electrostatic interaction between the positively charged capping agent of the rods and the negative charge of BSA. The size distribution of the BIO-NRCs has been investigated at room temperature by DLS measurements carried out at regular intervals (5 min) over a time period of 25 min (data not shown). No substantial changes of the mean hydrodynamic radius are detected. At 60 °C the size distribution of these objects shows an evident increment of the mean hydrodynamic radius to ca. 100 nm already ten minutes after stabilizing the temperature (c, black symbols), with no changes detected afterwards, proving that the temperature increase fosters the formation of larger BIO-NRCs.

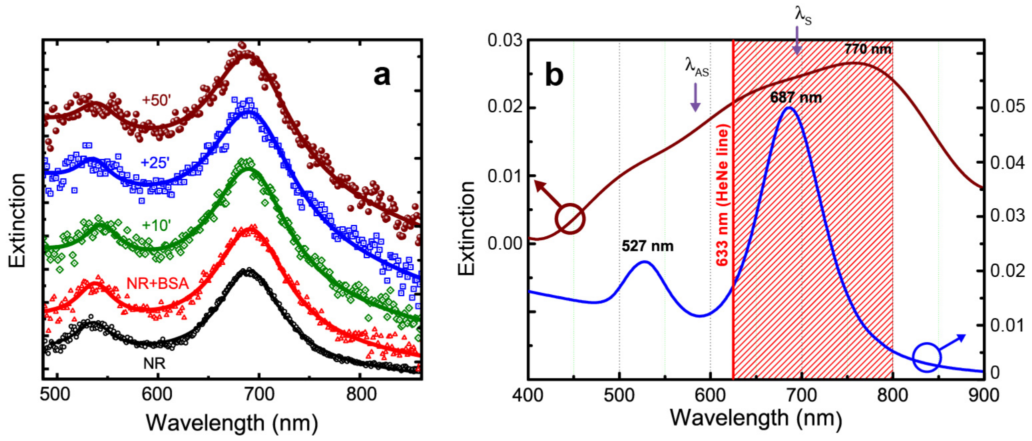

Figure S2: Extinction spectra. (a) Extinction spectra carried out on individual NRs, as purchased (black) and on NRs mixed with BSA in PBS at increasing times elapses from the mixing, on an total period of 50 min (colored data). Symbols refer to the experimental data, lines are fits using a Lorentz model. NRs as purchased display two distinct LSPR peaks at 527 nm and 687 nm, related to the short axis and long axis the dipolar resonances. Upon mixing with BSA the signal is still dominated by the resonances of the single NRs, slightly red-shifted (1 - 1.5 nm) and broadened (ca. 20 nm), probably a consequence of the change of local dielectric constant. Optically induced aggregation of the BIO-NRCs causes (b, brown line) a much larger broadening of the resonance with a red shift towards 770 nm with respect to the NRs as purchased (b, blue line). [2,3] The aggregates are SERS-active and optically resonant at both the laser (vertical red line) and the Raman scattering energy (dashed red box). Extinction on the individual NRs is acquired with a Perkin Elmer Lamda-20 UV-VIS spectrometer. Extinction on the NRs protein complexes is acquired by using a Xe lamp white light source, positioned below the liquid cell, collecting the light transmitted through the aggregate with the same objective used to induce it. The arrows indicate the wavelengths of the Stokes (λ_S) and anti-Stokes (λ_{AS}) photons corresponding to the 1390 cm^{-1} vibration, used for temperature estimation in SERS of BSA.

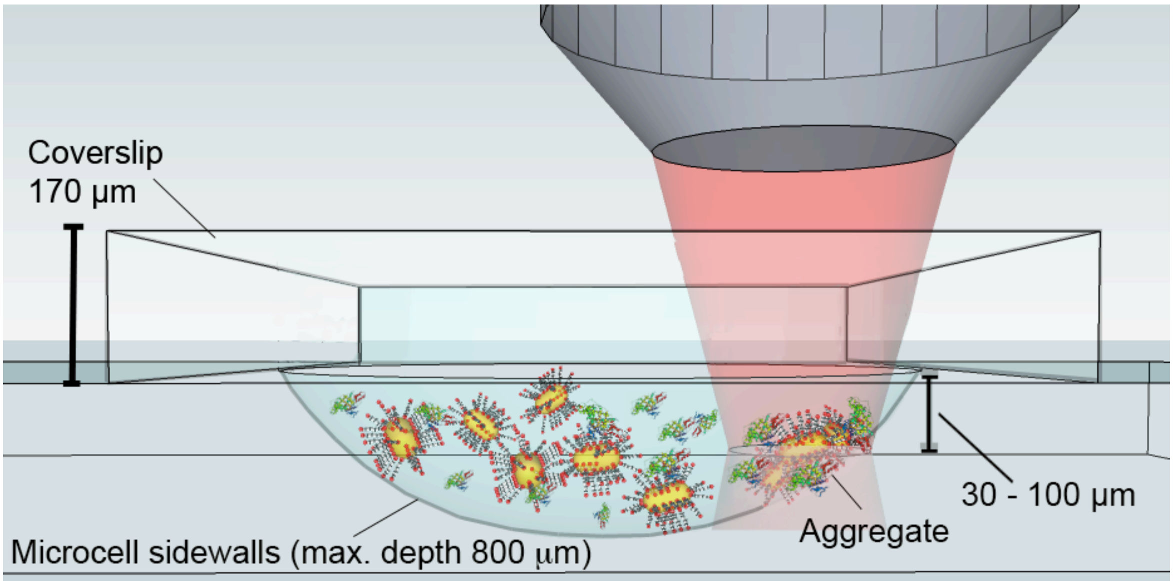

Figure S3: Sketch of the LIQUISOR geometry. An efficient aggregation of the BIO-NRCs complexes is obtained only when the laser spot is focused close to the bottom of the microcell. When this condition is met the BIO-NRCs, pushed down by the radiation pressure, are conveyed towards the bottom of the microcell where they stick and aggregate on the glass walls. Since the depth of the coverslip has a thickness of 170 µm and the working distance (WD) of the microscope objectives used for the experiments (100X, NA = 0.90, or 50X, NA = 0.75) is limited to 200 – 380 µm, the laser spot must be focused close to the lateral rims of the cell, where the hemispherical cell shape allows one to confine the rods in the 30 – 100 µm region between the coverslip bottom face and the sidewall. The achievement of this experimental condition is crucial to trigger the aggregation effect. Optical aggregation can also be carried out with long working distance objectives (50X, NA 0.5, WD 10.6mm), allowing more freedom of operation. In this case the laser spot must be focused slightly below the bottom face of the microcell in order to trigger the aggregation.

Supplementary Movie 1

NRs-BSA complexes passing through the focal laser spot scatter light as they are pushed away from the laser spot in the laser propagation direction, towards the bottom of the microcell. No 3D trapping occurs because at the laser wavelength employed (632.8 nm) the gradient forces, responsible for optical trapping, can be neglected with respect to the repulsive scattering forces. [4]

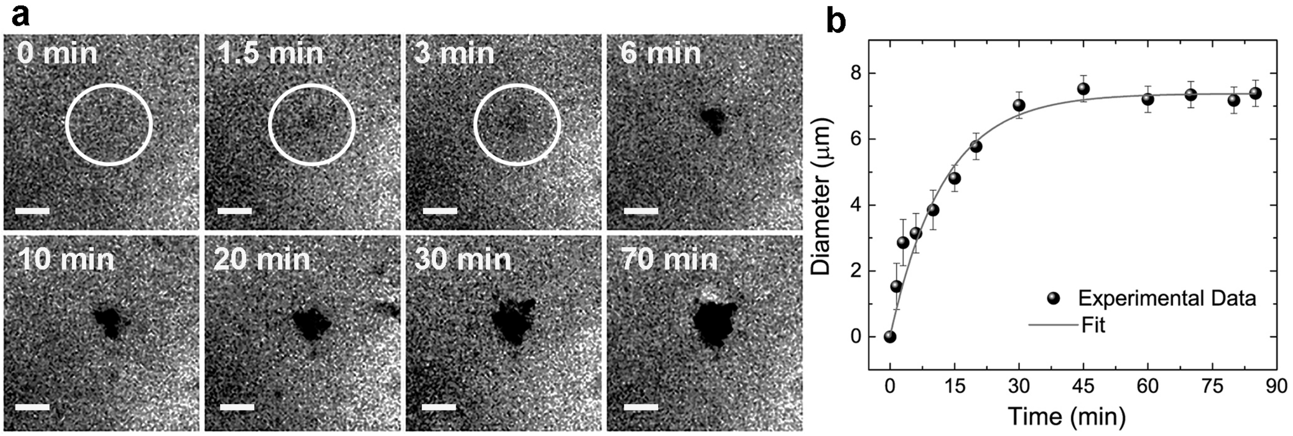

Figure S4: Dynamic aggregate growth under laser irradiation. (a) Time series images showing the formation of an aggregate under laser irradiation of gold NRs in BSA. Scale bar is 2.5 μm. Experiments are carried out on an XploRA PLUS spectrometer with a 50X long working distance microscope objective (Olympus LMPlanFl, NA 0.5, WD = 10.6mm). Optical images are acquired monitoring the light transmitted from a white lamp placed underneath the sample after repeated periods of laser irradiation (638nm, 13 mW). (b) Variation of the aggregate diameter as a function of time (solid symbols). Data are fitted (solid line) with a function $D(t) = A[1 - \exp(-t/t_0)]$ indicating an initial phase in which the aggregate diameter increases linearly, followed by a saturation regime. Best fit parameters are $A = (7.2 \pm 0.2)$ μm and $t_0 = (13 \pm 1)$ min.

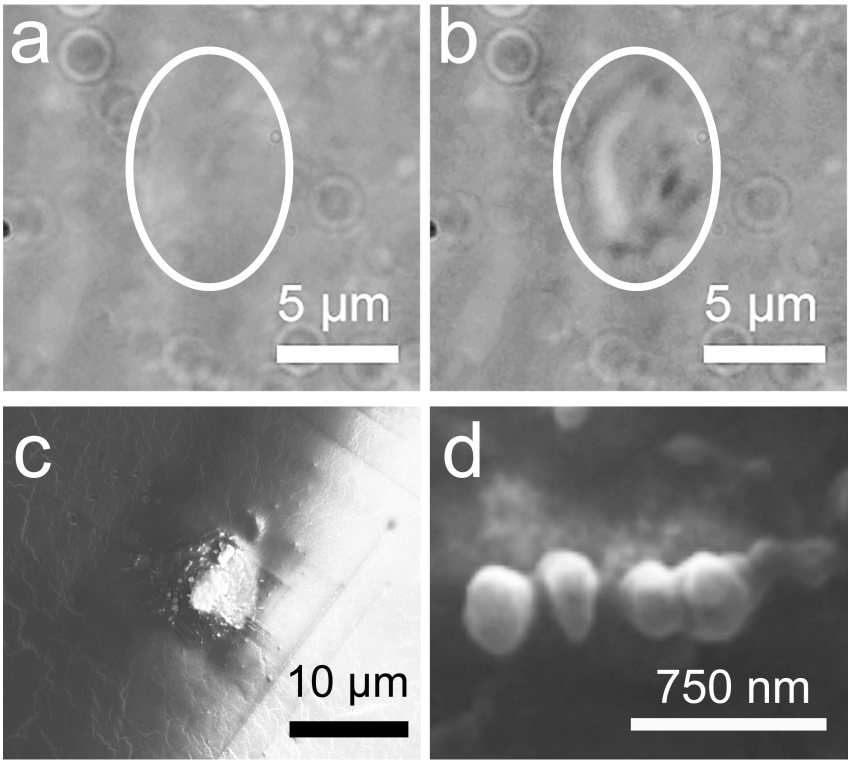

Figure S5: Bright field optical microscopy images before (a) and after the formation of a large aggregate (b) of NRs in BSA on the side of the glass microcell. (c) Scanning Electron Microscopy and (d) close-up on the same aggregate. In order to find the aggregate after transfer to SEM we have: (1) confined the aggregation process in a marked zone of the glass cell so to confine further SEM analysis within a few mm^2 of area; (2) after removing the glass coverslip we pipetted away the excess liquid and absorb the remaining liquid carefully; (3) we looked for isolated, symmetric objects, not surrounded by other sparse nanorods aggregates on distances of some tens of microns.

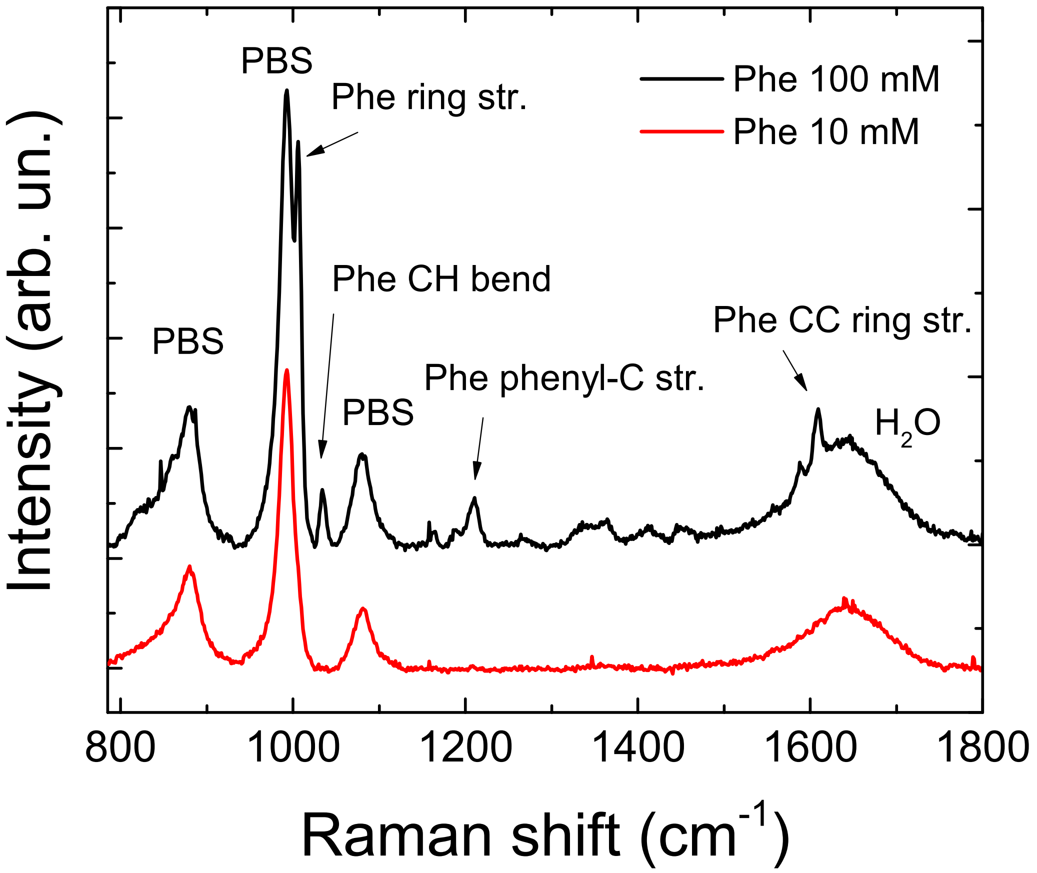

Figure S6: Raman spectra of Phe in PBS solution at concentrations of 10mM (red line) and 100 mM (black line). The most intense Raman vibrations of Phe are visible at concentrations of 100 mM. The modes assignment is carried out according to refs.[5, 6] At concentrations of 10 mM only the PBS peaks are detected. Laser power 6.7 mW, integration time 300 s.

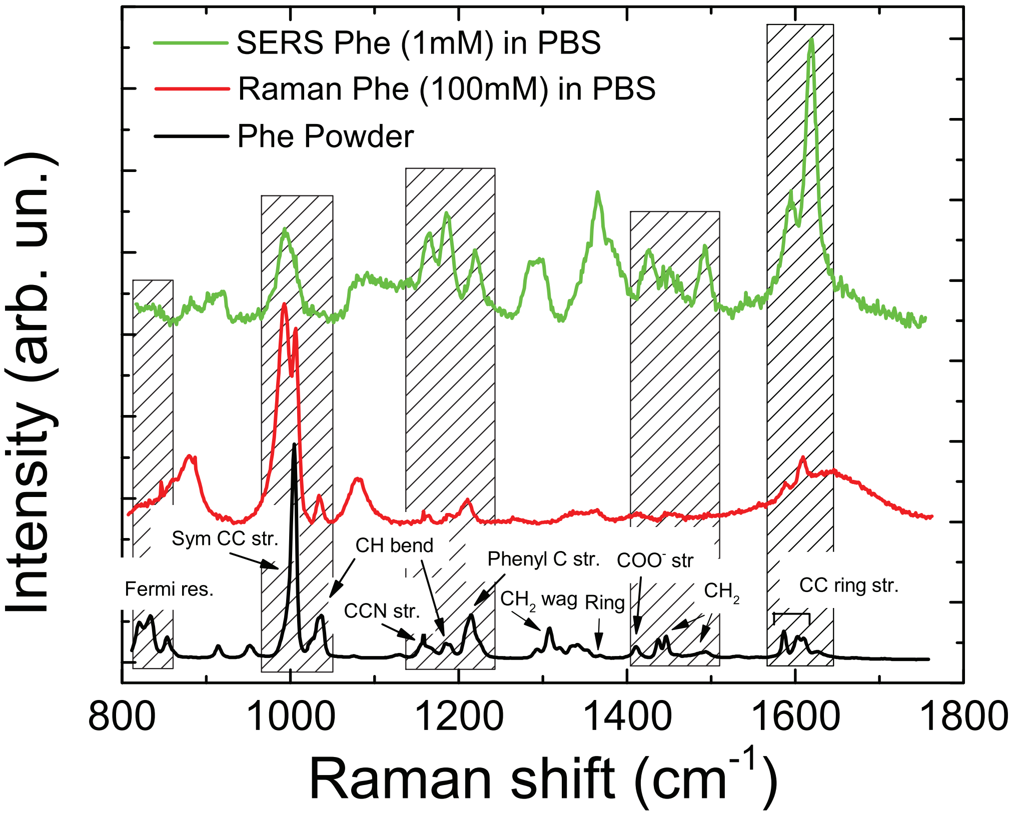

Figure S7: Comparison between Raman spectra of Phe powder (black line), Raman of Phe in PBS solution (100 mM, red line) and SERS of Phe in PBS solution (1 mM, red line). The modes assignment is carried out according to references [5,6,7,8] (see Supplementary Table S1). The dashed boxes are used to highlight the correspondence between the vibrational modes detected using the different samples/techniques.

Supplementary Table S1

Table S1: **Vibrational modes of Phe measured in solid, in PBS solution (100 mM) and by the LIQUISOR method (1 mM).** Comparison with SERS measurements carried out in the literature is given. Modes assignment is carried out following the literature.

Peaks position (cm^{-1})				
**This work**			**Literature**	
**Phe Solid**	**Phe in PBS**	**Phe SERS**	**Phe SERS on Au, Ag**	**Modes Assignment**
820, 833, 853	815-835, 859	815-835	829(10), 851(7)	Fermi resonance between ring breathing and out of plane ring bend overtone.[6,8]
-	880	881		PBS[9]
914, 952	-	918		CC stretching[6]
-	993	993		PBS[9]
1004	1006	1005	1001(8), 1005,(7) 1003.4(10)	Symmetric CC ring stretching[5,6,7,8,11]
1035	1034	-	1034(7,8), 1032.9(10)	In-plane CH bending5,[6,7,8]
-	1080	1081		PBS[9]
1158	1158, 1164	1164	1155(10)	In-plane CH bending, ring CH bending[6]
1184	1186	1185	1185(10)	Combination of in-plane CH bending[5,6,7]
1215	1210	1218	1204(10)	Phenyl-C stretching[5,6,7,11]
1294	-	-	1297[10]	Not assigned[6]
1308	-	1280 - 1300		CH_2 wag[8] or Ring stretching[6]
1334 - 1340	-	-		Observed but not assigned[6]
1368	1364	1365		Not assigned
1410	-	-	1411(8)	COO^- stretching[7,8] or Ring stretching/CH bending[6]

1436, 1446	1445	1424, 1452	1446.8[10]	CH_2 scissor[6,8]
1494	-	1492		Not Assigned (probably CH or CH_2 bending)
1586	1588	1595	1586[(8)], 1598.2[(10)]	In plane ring stretching[5,6,7,8,11]
1601, 1610	1610	1616	1602[(8)], 1603[(7)], 1643[(7)]	In plane ring stretching[5,6,7,8,11]
-	1640 (band)	-		Water OH bending[12]

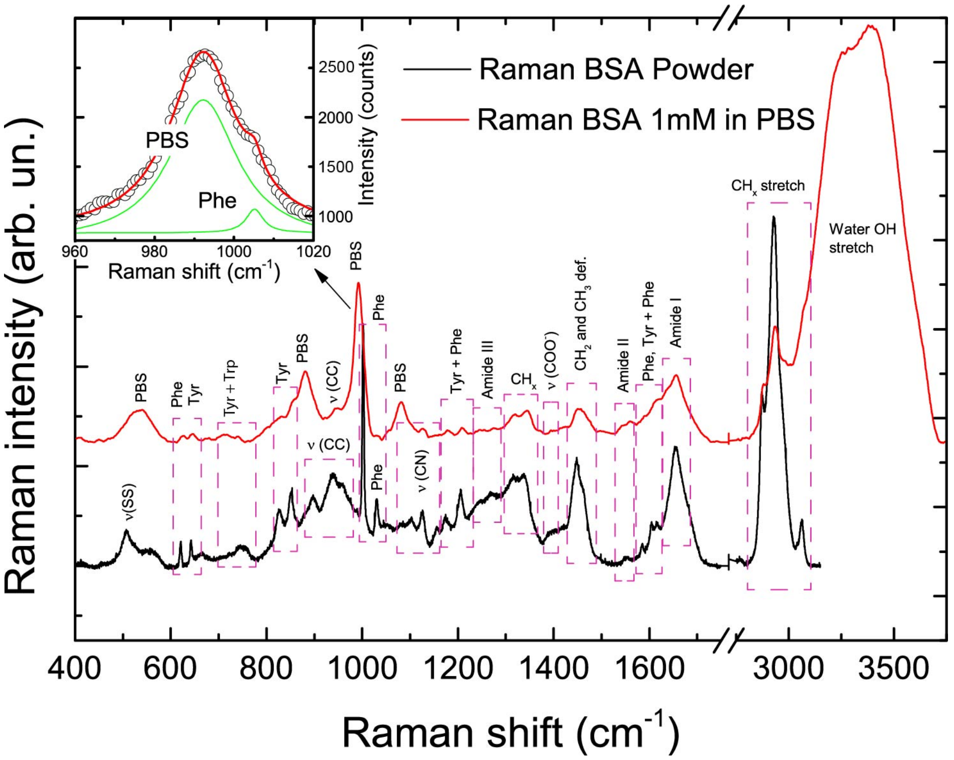

Figure S8: (red line) Solution phase Raman spectrum of BSA (1mM) in PBS compared to the Raman spectrum of BSA in powder state (black line). The modes assignment is carried out following the references enlisted in Supplementary Table S2. The inset displays the liquid phase spectrum of BSA in the 960 – 1020 cm^{-1} range (hollow symbols), highlighting the Phe peak at 1006 cm^{-1} convolved to the PBS intense vibration at 992 cm^{-1}. A double Lorentzian fitting (red line) allows to separate the two peaks and retrieve both peak positions and intensity. Laser Power 6.7 mW. Integration time 30 s.

Supplementary Table S2

Table S2: **Vibrational modes of BSA measured in PBS solution (1 mM), in powder state and through the LIQUISOR method (100 µM).** Mode assignment is carried out based on the literature data and, when no data are found, based on the Raman spectra carried out on aromatic amino acids in powder state (blue boxes in Supplementary Fig. S9). Phe: Phenylalanine. Tyr: Tyrosine. Trp: Tryptophan.

**Observed in this work**			
Raman of BSA in PBS (1 mM)	Raman of BSA in powder state	SERS of BSA in PBS (100 µM)	Tentative mode assignment of the Raman modes
-	507	506, 520, 550	SS stretching[13,14,15]
533	-	-	PBS watery solution
-	-	600	Phe COO^- deformation [6] (Fig. S7)
624	621	-	Phe ring (C-C) deformation [13]
644	642	646	Tyr ring (C-C) deformation[13]
712, 740	719, 742	713, 737	Observed but not assigned in ref.[14] Tyr (Fig. S7)
-	755	-	Trp[13]
830,855	826, 852	834, 854	Tyr Fermi doublet[13,14]
-	-	862	Tyr (Fig. S7)
881	-	-	PBS
-	898, 938	-	CC stretching[13] or CCN_{sym} stretching[14]
950	955	955 (shoulder)	CC stretching[13]
992	-	-	PBS
1005, 1032	1002, 1033	1004, 1039	Phe ring breathing[13,14,16]
-	-	1073	Trp (Fig. S7)
1083	-	-	PBS
1126	1082, 1102, 1126, 1156	1130, 1158	CN stretching[13,14]
1177, 1209	1174, 1205	1204	Tyr + Phe[13,14]

1243, 1275	1244, 1268	1239,1274	Amide III[13,14,16]
1318, 1346	1315, 1337	1314	CH_2 twisting[13] or CH bending[14]
-	-	1371	Tyr (Fig. S7)
1394	1398	1395	Aromatic amino acids COO^- stretching[13,14, 17]
1453, 1472	1448, 1460	1456	CH_2 and CH_3 scissoring[13,14,16]
		1497	Phe (Fig. S7)
1548	1550	-	Amide II[13]
1558	-	1565	Trp (Fig. S7)
1588	1585	1588, 1592	Phe[13,14]
1618	1605, 1618	1605, 1615	Tyr + Phe[13,14]
1655 (band)	1656 (band)	1650	Amide I [13,14]
2880, 2931, 3070	2872, 2927, 3062	2820 – 3000 (band), 3066	CH stretching, Tyr + Phe (Fig. S7)[18]
3250, 3389 (band)	-	3250, 3390 (band)	Water OH stretching[12]

Supplementary Table S3

Table S3. **SERS peaks positions of BSA reported in the literature and comparison with our findings.** Highlighted in bold are the modes which have been observed more frequently and are suggested as marker bands of BSA in SERS experiments.

This work	Ref. [10]			Ref. [8]	Ref.[19]	Ref.[20]	Ref.[21]	Ref. [22]	Ref. [23]	Ref. [16]	Ref. [24]	Ref. [25]
SERS Au	TERS	SERS Au	SERS Ag	SERS Ag	SERS Ag	SERS Ag	SERS Ag	SERS Au	SERS GaN	SERS Ag	SERS Au	SERS Au
506, 520, 550							503				520	
-												
600										605		
**-**	**619**	**621**	**618**	**614**			**617**		**620**			
646				640			640				637	
					665							
-	695		695	691								
713, 737	709	709	709								704	
				747								
-	761		760		766				768	765		
**834, 854**	**829, 853**	**853**		**829, 840**		**827, 851**	**825, 847**		**852**			
862												
-				872								
				920						903		
-	937		931									
							947					
955 (shoulder)												
**1004, 1039**	**1004, 1028**	**1003**	**1004**	**998, 1026**	**1006**	**1002, 1031**	**1000, 1029**		**1001**	**1024**	**1005**	**1006**
1073						1073					1079	
-				1100						1118		
				1142								
1130, 1158	1129		1127					1145				

-				1177	1176				1174	1173		
1204				1211	1216	1206			1211			
**1239, 1274**	**1267**		**1265**	**1242, 1272**	**1284**	**1288**			**1291**		**1256**	**1252**
1314				1340		1337				1304		
					1331							
				1356				1351		1357		
1371												
1395						1403			1405			
**1456**	**1450**			**1451, 1476**		**1448**	**1441**		**1442**		**1446**	**1454**
1497												
-	1507									1504		
											1520	
					1555							
1565												
							1575			1570		
**1588**					**1584**	**1584**		**1580**	**1584**		**1584**	
**1595, 1605**	**1603**	**1603**	**1604**	**1605**	**1605**	**1605**		**1615**	**1605**	**1615**		
**1650**				**1643, 1665**	**1658**	**1655**	**1666**		**1649**	**1645**		**1658**
2820 – 3000 (band), 3066												

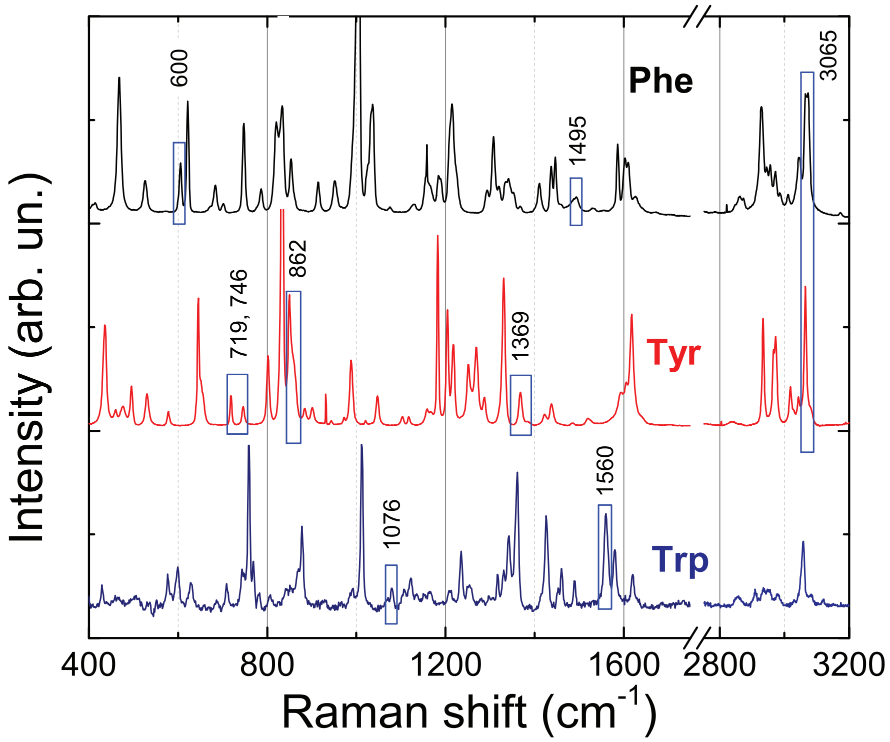

Figure S9: **Raman spectra of the aromatic amino acids in powder state.** Spectra are shown in the low (400 – 1750 cm^{-1}) and high frequency (2750 – 3200 cm^{-1}) range. Phenylalanine (black), Tyrosine (green), Tryptophan (blue). The blue boxes highlight the Raman modes that are enhanced in the SERS spectra of BSA but whose assignment has not been found in the literature. Laser wavelength 633 nm, power 1 mW, integration time 10 s.

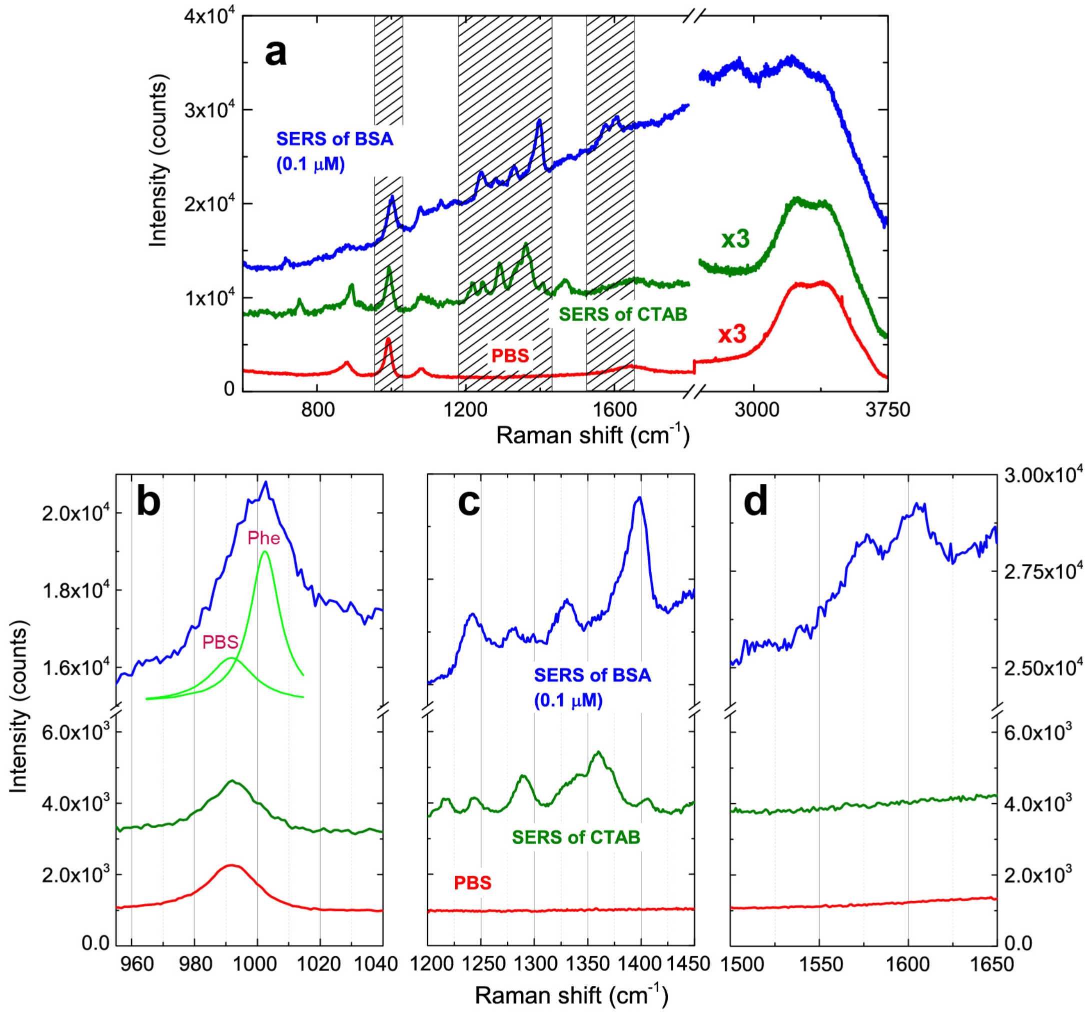

Figure S10: Control experiments on BSA. (a) Comparison between Raman spectrum of PBS (red), SERS spectrum of CTAB induced by NRs aggregation (green line) and SERS of BSA 100 nM in PBS (blue line) obtained by LIQUISOR method in the same experimental conditions. The CTAB and PBS SERS intensities are multiplied by 3 for comparison purposes. The dashed areas highlight the spectral zones in which the zooms displayed in (b, c, d) have been carried out. In (b, c, d) the intensities are reported as measured, without rescaling. The PBS signal and the SERS contribution from the CTAB are spectrally different and less intense than SERS from BSA at 100 nM. According to the literature,[26],[27] a contribution of CTAB cannot be excluded in the high frequency CH stretching modes up to 2950 cm^{-1}, while no signal has to be expected from the surfactant at 3066 cm^{-1}.

Supplementary Note 2

A similar enhancement of the COO^- symmetric stretching has been observed by Kaminska et al. [28,29] in the interaction between bovine pancreatic trypsin inhibitor (BPTI) and CTAB-protected gold nanoparticles deposited on functionalized silicon surface. The intensity ratio I_{855}/I_{830} of the doublet of tyrosyl residues at 855 and 830 cm^{-1}, which origins from Fermi resonances between the symmetric ring-breathing fundamental and the overtone of the non planar ring vibration, is a good indicator of the nature of the hydrogen bonding of the phenolic hydroxyl,[30] in particular the upper frequency peak becomes stronger when the OH group is weakly H bonded to a negative acceptor such as the COO^-. In the Raman spectrum of the native BSA at pH 7 the typical Fermi doublet intensity ratio is 10:9, while in SERS spectra we notice a strong enhancement of the higher frequency peak (854 cm^{-1}) with an estimated intensity ratio of 10:1, that confirms the conformational change of proteins with the tyrosyl residues and the COO^- groups not H-bonded between them and interacting with the CTAB chains.

Supplementary Note 3

According to the E^4 model, the SERS intensity I_{SERS} can be expressed as[31,32]

$$I_{\mathrm{SERS}} \propto \Gamma_{\mathrm{exc}}^2(\lambda_L)\times\Gamma_{\mathrm{rad}}^2(\lambda_R)\times I_L \qquad \text{(Eq. S2)}$$

where Γ_{exc} (λ_L) and Γ_{rad} (λ_R) are, respectively, the enhancement factors relative to the excitation field at wavelength λ_L and to the re-radiated Raman field at wavelength λ_R. I_L is the incident field intensity. Le Ru et al. assume that $\Gamma^2(\lambda) \propto Q_e(\lambda)$, where $Q_e(\lambda)$ is the extinction spectrum, for both the excitation and the re-radiation.[33] Experiments on near-field coupled nanoparticles, in which the excitation wavelength is scanned while recording the intensity of a Raman mode, show that the excitation enhancement factor Γ_{exc} (λ_L) is red-shifted and uncorrelated to the extinction (see ref. [34] and references therein). In particular, Γ_{exc} (λ_L) is shown to strongly increase (ca. 2 orders of magnitude) when the excitation varies from the visible (600nm) to the NIR (850nm). Experiments more focused on the re-radiation enhancement Γ_{rad} (λ_R) at various excitation wavelengths,[35] show that at 633nm the re-radiation enhancement of the Stokes modes is almost constant in the range from 450 to 1630 cm^{-1}, while strong variations are observed for excitations at lower wavelengths (515, 532nm).

Temperature is estimated from the anti-Stokes/Stokes amplification ratio $\eta = I_{AS}/I_S$, that is expected to be influenced by the nanoparticles re-radiation properties. The anti-Stokes photons at wavelength λ_{AS} will be amplified by a factor $\Gamma_{\mathrm{rad}}^2(\lambda_{AS})$, whereas the Stokes ones at wavelength λ_S will be amplified by a factor $\Gamma_{\mathrm{rad}}^2(\lambda_S)$.

In general, we expect that the anti-Stokes/Stokes intensity ratio in SERS will be modified as

$$\frac{I_{\mathrm{AS}}^{\mathrm{SERS}}}{I_{\mathrm{S}}^{\mathrm{SERS}}} = \frac{\Gamma_{\mathrm{rad}}^2(\lambda_{AS})}{\Gamma_{\mathrm{rad}}^2(\lambda_S)}\frac{I_{AS}}{I_S} \qquad \text{(Eq. S3)}$$

In Le Ru's hypothesis $\Gamma_{\mathrm{rad}}^2(\lambda_{AS}) \propto Q_e(\lambda_{AS})$, $\Gamma_{\mathrm{rad}}^2(\lambda_S) \propto Q_e(\lambda_S)$. In most of the reported experimental cases $\Gamma_{\mathrm{rad}}^2(\lambda_{AS})/\Gamma_{\mathrm{rad}}^2(\lambda_S) < 1$, i.e. a lower re-radiation enhancement of the anti-

Stokes modes at λ_{AS} is found with respect to the Stokes ones at λ_S. This causes a systematic underestimate of the sample temperature. In order to compensate such effect we can retrieve the "true" I_{AS}/I_S values from the SERS data using Eq. S3, i.e. multiplying the raw SERS ratio η by a factor $\Gamma^2_{\mathrm{rad}}(\lambda_S)/\Gamma^2_{\mathrm{rad}}(\lambda_{AS})$.

From the quantitative point of view, if the relative correction to the anti-Stokes/Stokes ratio η is small, i.e. $\Delta\eta/\eta < 1$, the expected relative temperature change is

$$\Delta T/T = \frac{kT}{E_{\mathrm{R}}}\Delta\eta/\eta \qquad \text{(Eq. S4)}$$

For the SERS mode of BSA at 1390 cm^{-1} (172 meV) the change is $\Delta T/T \sim 4.5\%$, while for the mode at 600 cm^{-1} $\Delta T/T \sim 10.5\%$ (T is the absolute temperature in degrees Kelvin).

In those cases in which the correction to the anti-Stokes/Stokes ratio is expected to be relevant and to change abruptly with the wavelength, as in the case reported by ref.[34] (i.e. up to orders of magnitude), the temperature calculated from the raw SERS data would give values well below ambient temperature (i.e. unphysical in typical experimental conditions), due to the strong over-amplification of the Stokes peaks. In addition, using the EF wavelength dependence in ref.[34], differences as large as 50 °C would be expected if the temperature is calculated at 600 cm^{-1} or at 1390 cm^{-1}, due to the fact that the over-amplification of the Stokes intensity grows with the wavelength shift between Stokes and anti-Stokes peaks which, in turn, increases with the vibration energy.

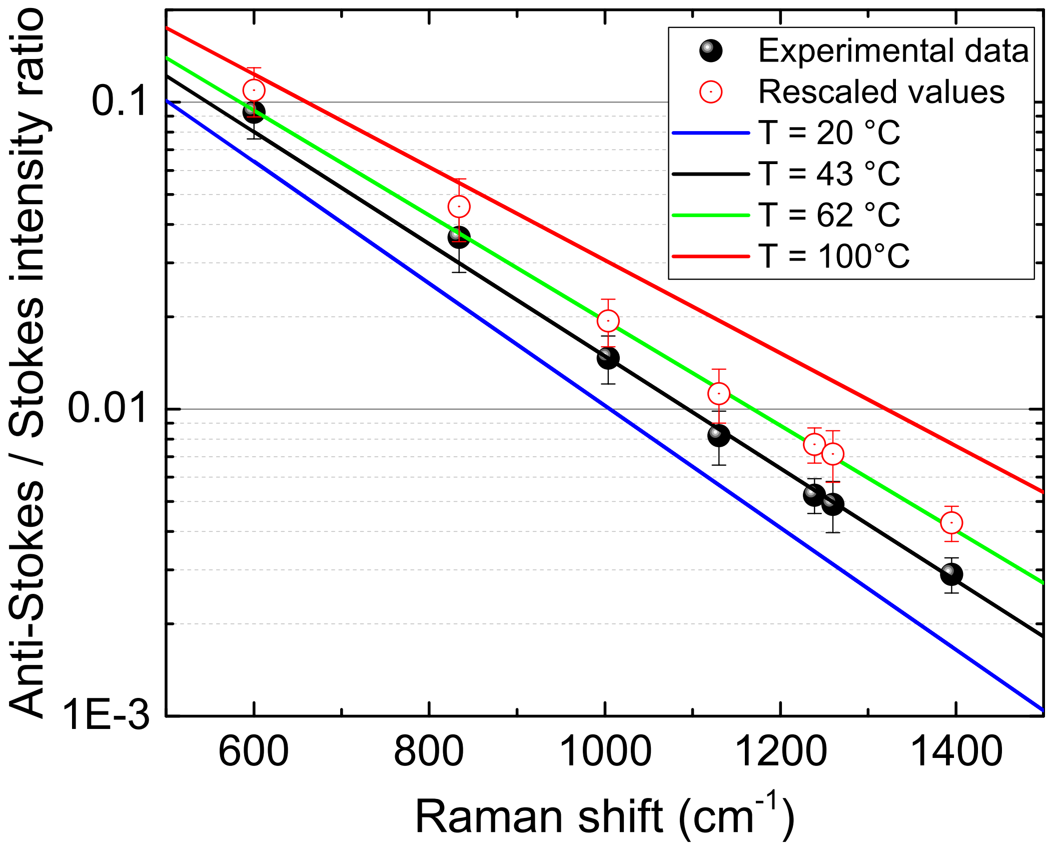

Figure S11: (black symbols) Anti-Stokes to Stokes intensity ratio measured on the different SERS peaks of BSA, experimental raw data. (black line) Best fit of the raw data yields a temperature of 43 °C. (red symbols) Experimental data rescaled on the extinction profile (Figure S3, brown line) using Eq. S3. (green line) Best fit of the rescaled data yields a temperature of 62 °C. Blue and red lines highlight, respectively, the Anti-Stokes to Stokes intensity ratio expected at room temperature (20 °C) and at the water boiling point (100 °C) and are guides-to-the-eye of the minimum and maximum temperatures expected in our experiments.

Supplementary Note 4

The SERS peaks at 506, 520 and 550 cm^{-1} are ascribed to the change in the S-S stretching mode frequency due to the three different configurations of the dihedral angle between C_β-S-S and S-S-C_β planes, gauche-gauche-gauche (*ggg*), gauche-gauche-trans (*ggt*) and trans-gauche-trans (*tgt*) respectively,[17] with a content estimated by the Raman analysis of 38%, 56% and 6%. The higher content of *ggt* form and an intensity ratio $I_{(506)}/I_{(520)}$ of 0.7 is an indication of the BSA in solution in the native form [17,36] despite the local temperature during the SERS experiment is close to that of protein conformational changes.[14]

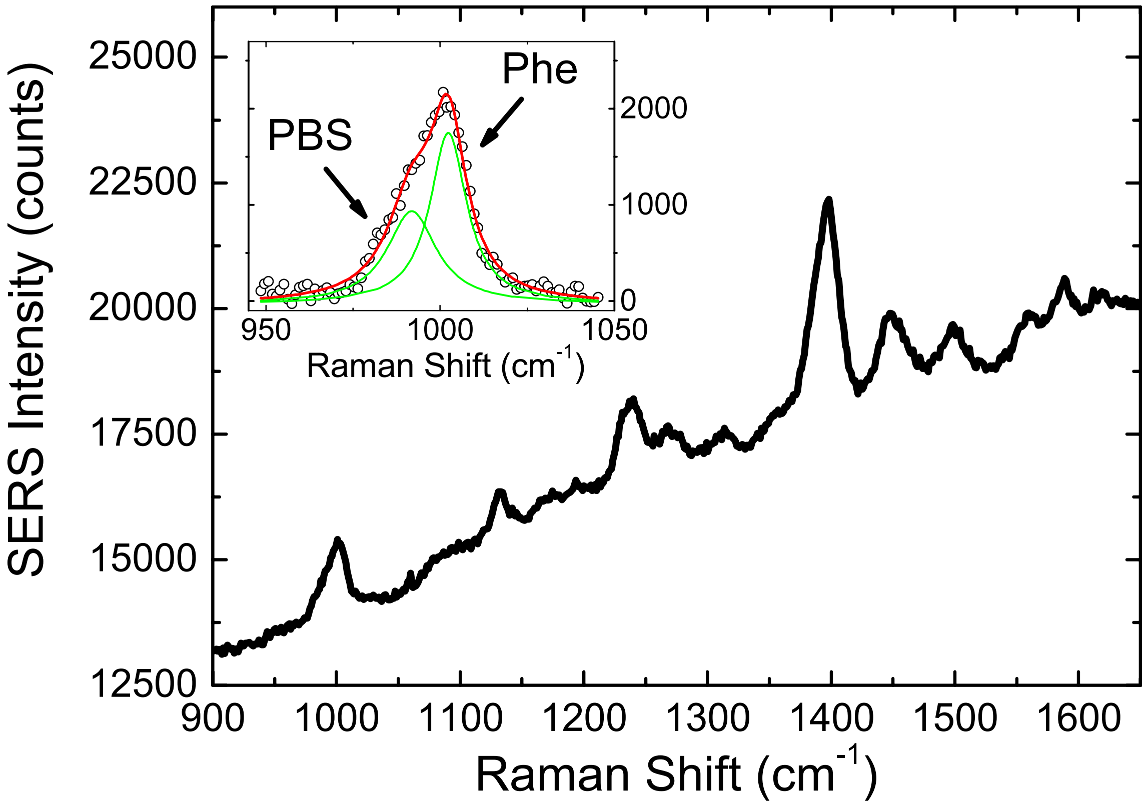

Figure S12: SERS spectrum of BSA at 50nM concentration probed by the LIQUISOR method. The inset shows a zoom in the 950 – 1050 cm^{-1} range. The fit of the experimental data as the sum of two Lorentzian functions (green line, hollow symbols and red lines, respectively) highlights the prevalence of the Phe peak at 1006 cm^{-1} with respect to the PBS vibration at 992 cm^{-1}. Laser Power 6.7 mW, microscope objective 100X. Integration time 20 s.

Supplementary Table S4

Table S4: Peaks positions measured in the Raman spectrum of Lys 10 mM in PBS liquid solution, in the Raman spectrum of Lys in powder state, in the SERS spectrum of Lys 1 μM in PBS liquid solution after formation of the SERS-active aggregate. Mode assignment is carried out based on the literature data.

**Observed in this work**			
Raman of LYS in PBS (10 mM)	Raman of LYS in powder state	SERS of LYS in PBS (1 μM)	Tentative mode assignment of the Raman modes
763	761		Trp[37]
-	832, 857	829, 855	Tyr Fermi doublet[13]
881	880	886	PBS + Trp[37]
900, 938	899, 935	-	CC Stretching[37, 38]
961	960	-	Not Assigned[37]
993	-	994	PBS
1008, 1033	1006, 1032	1004	Phe[37]
1015	1014	1022	Trp[37]
1082	-	1081	PBS
1077, 1107, 1131	1078, 1107, 1128	1116, 1142	C-N Stretching[37]
1159	1158	-	Not Assigned[37]
1177	1180	1175	Tyr[37]
1201, 1210	1210	1200	Tyr + Phe[37]
1240, 1260	1240, 1255	1246, 1268	Amide III[37]
1340, 1370	1340, 1363	1346, 1378	Trp[37]
-	1390	1395	Aromatic amino acids COO^- stretching[13]
1450	1448	1452	CH Deformation[37]
-	-	1493	Phe (Fig. S7) or His[37]
-	-	1540	Not assigned
1554, 1581	1553, 1581	1571	Trp[37]

1620	1608, 1621	1610,1625	Trp, Tyr + Phe[37]
1658	1659	1637	Amide I[37]

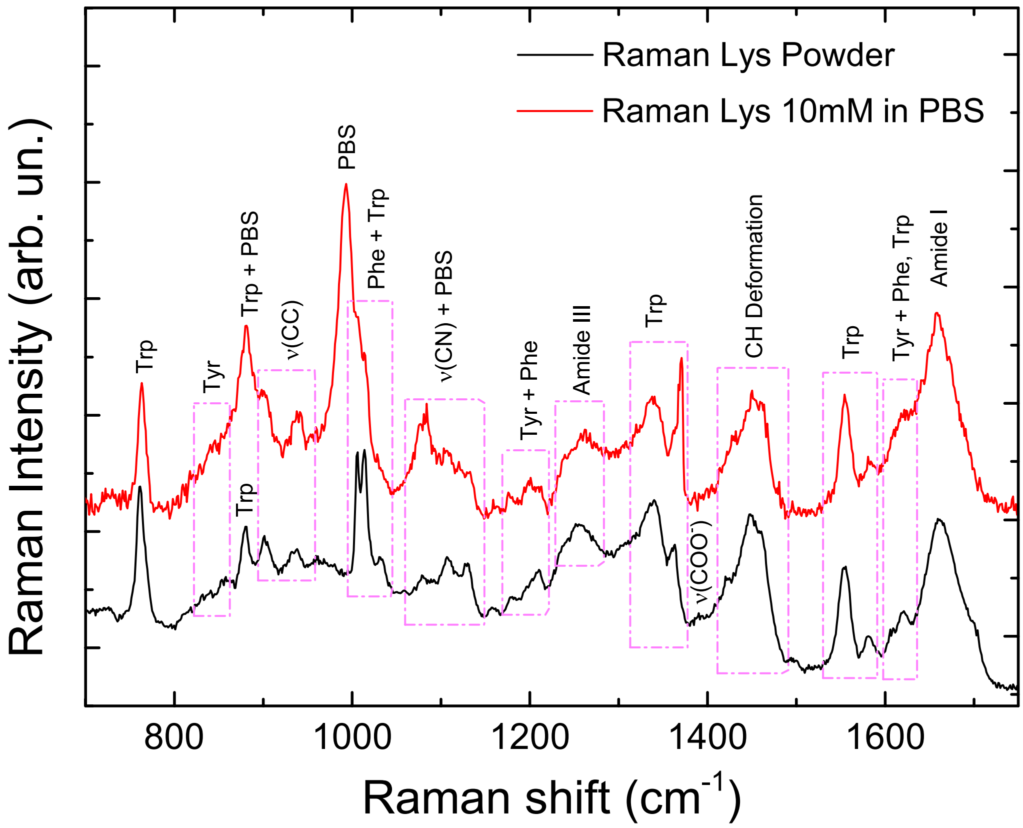

Figure S13: (red line) Solution phase Raman spectrum of Lys (10mM) in PBS compared to the Raman spectrum of Lys in powder state (black line). Laser Power 6.7 mW. Integration time 30s. The modes assignment is carried out following the references enlisted in **Supplementary Table S4**.

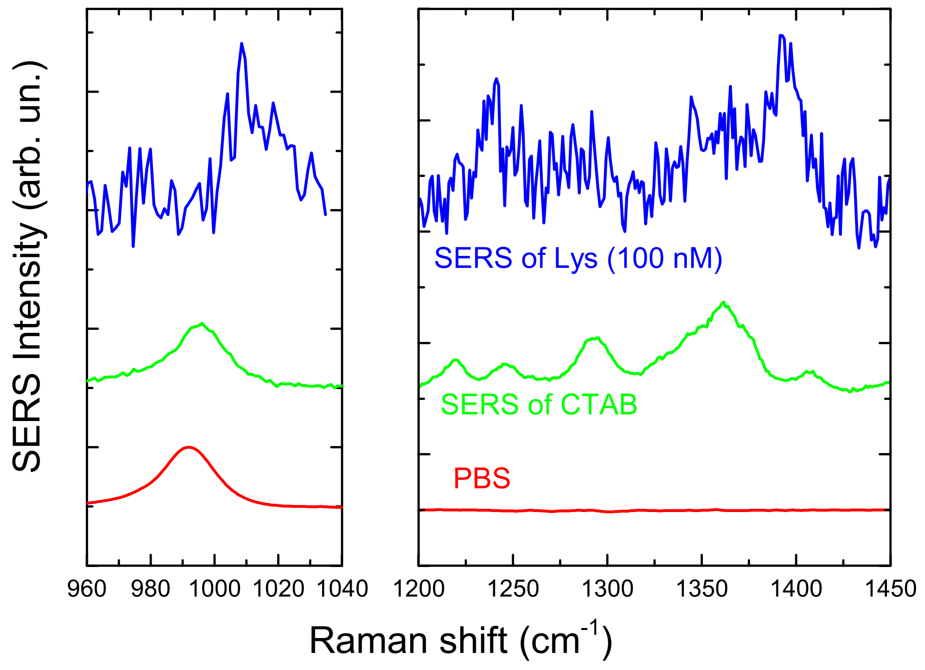

Figure S14 Comparison between Raman spectrum of PBS (red), SERS spectrum of CTAB induced by NRs aggregation (green line) and SERS of Lys 100 nM in PBS (blue line) obtained by LIQUISOR method in the same experimental conditions. The spectrum of Lys is plotted after subtraction of the PBS background signal.

References

[1] Villari, V. & Micali, N. Light Scattering as Spectroscopic Tool for the Study of Disperse Systems Useful in Pharmaceutical Sciences. *J. Pharm. Sci.* **97**, 1703-1730 (2008).

[2] Halas, N.J., Lal, S., Chang, W.S., Link, S. & Nordlander, P. Plasmons in Strongly Coupled Metallic Nanostructures. *Chem. Rev.* **111**, 3913–3961 (2011).

[3] Messina, E., et al. Manipulation and Raman Spectroscopy with Optically Trapped Metal Nanoparticles Obtained by Pulsed Laser Ablation in Liquids. *J. Phys. Chem. C* **115**, 5115-5122 (2011).

[4] Königer, A. & Köhler, W. Optical Funneling and Trapping of Gold Colloids in Convergent Laser Beams. *ACS Nano* **6**, 4400–4409 (2012).

[5] Asher, S. A., Ludwig, M., & Johnson, C. R.. UV resonance Raman excitation profiles of the aromatic amino acids. *J. Am. Chem. Soc.* **108**, 3186-3197 (1986).

[6] Zhu, G., Zhu, X., Fan, Q. & Wan, X. Raman spectra of amino acids and their aqueous solutions. *Spectrochim. Acta A* **78**, 1187-1195 (2011).

[7] Podstawa, E., Ozaki, Y. & Proniewicz, L. M. Part I: surface-enhanced raman spectroscopy investigation of amino acids and their homopeptides adsorbed in colloidal silver applied spectroscopy. *Appl. Spectrosc.* **58**, 570 – 580 (2004).

[8] Stewart, S., & Fredericks, P. M. Surface-enhanced Raman spectroscopy of amino acids adsorbed on an electrochemically prepared silver surface. *Spectrochim. Acta A* **55**, 1641-1660 (1999).

[9] David, C., et al. Raman and IR spectroscopy of manganese superoxide dismutase, a pathology biomarker. *Vib. Spectrosc.* **62,** 50-58 (2012).

[10] Blum, C., Schmid, T., Opilik, L., Weidmann, S., Fagerer, S. R., & Zenobi, R.. Understanding tip-enhanced Raman spectra of biological molecules: a combined Raman, SERS and TERS study. *J. Raman Spectrosc.* **43**, 1895-1904 (2012).

[11] Fischer, W. B., & Eysel, H. H. Polarized Raman spectra and intensities of aromatic amino acids phenylalanine, tyrosine and tryptophan. *Spectrochim. Acta A* **48**, 725-732 (1992).

[12] Carey, D. M., & Korenowski, G. M. Measurement of the Raman spectrum of liquid water. *J. Chem. Phys.* **108**, 2669 (1998).

[13] Chen, M.C. & Lord, R.C. Laser-Excited Raman Spectroscopy of Biomolecules. VIII. Conformational Study of Bovine Serum Albumin. *J. Am. Chem. Soc.* **98**, 990-992 (1976).

[14] Lin, V.J.C. & Koenig, J.L. Raman Studies of Bovine Serum Albumin, *Biopolymers* **15**, 203-218 (1976).

[15] Nakamura, K., Era, S., Ozaki, Y., Sogami, M., Hayashi, T., & Murakami, M. Conformational changes in seventeen cystine disulfide bridges of bovine serum albumin proved by Raman spectroscopy. *FEBS Lett.* **417**, 375-378 (1997).

[16] Cavalu, S., Cinta-Pinzaru, S., Leopold, N. & Kiefer, W. Raman and Surface Enhanced Raman Spectroscopy of 2,2,5,5-Tetramethyl-3-pyrrolin-1-yloxy-3-carboxamide Labeled Proteins: Bovine Serum Albumin and Cytochrome c. *Biopolymer* **62**, 341 (2001)

[17] Navarra, G., Tinti, A., Leone, M., Militello, V. & Torreggiani, A. Influence of metal ions on thermal aggregation of bovine serum albumin: Aggregation kinetics and structural changes. *J. Inorg. Biochem.* **103**, 1729–1738 (2009).

[18] Socrates, G. *Infrared and Raman Characteristic Group Frequencies: Tables and Charts*, 3rd Edition (John Wiley & Sons, 2004).

[19] Maofeng, Z., et al. Rapid, large-scale, sonochemical synthesis of 3D nanotextured silver microflowers as highly efficient SERS substrates. *J. Mater. Chem.* **21**, 18817-18824 (2011).

[20] Han, X. X., et al. Analytical technique for label-free multi-protein detection based on Western blot and surface-enhanced Raman scattering. *Anal. Chem.* **80**, 2799-2804 (2008).

[21] Kahraman, M., Sur, I. & Culha, M. Label-free detection of proteins from self-assembled protein-silver nanoparticle structures using surface-enhanced raman scattering. *Anal. Chem.* **82**, 7596-7602 (2010).

[22] Jianhua, Z., et al. Convenient formation of nanoparticle aggregates on microfluidic chips for highly sensitive SERS detection of biomolecules. *Anal. Bioanal. Chem.* **402**, 1601-1609 (2012).

[23] Kamińska, A., et al. Highly reproducible, stable and multiply regenerated surface-enhanced Raman scattering substrate for biomedical applications. *J. Mater. Chem.* **21**, 8662-8669 (2011).

[24] Iosin, M., et al. Study of protein–gold nanoparticle conjugates by fluorescence and surface-enhanced Raman scattering. *J. Mol. Struct.* 924, 196-200 (2009).

[25] David, C. et al. SERS detection of biomolecules using lithographed nanoparticles towards a reproducible SERS biosensor." *Nanotechnology* **21**, 475501 (2010).

[26] Sun, S., Birke, R. L., & Lombardi, J. R. Surface-enhanced Raman spectroscopy of surfactants on silver electrodes. *J. Phys. Chem.* **94**, 2005-2010 (1990).

[27] Dendramis, A. L., Schwinn, E. W., & Sperline, R. P. A Surface-Enhanced Raman scattering study of CTAB adsorption on copper. *Surf. Sci.* ***134***, 675-688 (1983).

[28] Kaminska, A., et al., Chemically bound gold nanoparticle arrays on silicon: assembly, properties and SERS study of protein interactions. *Phys. Chem. Chem. Phys.* **10**, 4172-4180 (2008).

[29] Kaminska, A., Forster, R. J., & Kayes, T. E., The impact of adsorption of bovine pancreatic trypsin inhibitor on CTAB-protected gold nanoparticle arrays: a Raman spectroscopic comparison with solution denaturation. *J. Raman Spectrosc.* **41**, 130-135 (2010).

[30] Siamwiza M.N. *et al.* Interpretation of the Doublet at 850 and 830 cm^{-1} in the Raman Spectra of the Tyrosyl residues in proteins and certain Model Compounds. *Biochem.* **14**, 4870-4876 (1975).

[31] Le Ru, E. C. & Etchegoin, P. G. Rigorous justification of the$|E|^4$ enhancement factor in surface enhanced Raman spectroscopy. *Chem. Phys. Lett.* **423**, 63-66 (2006)

[32] Alonso-González, P. et al. Resolving the electromagnetic mechanism of surface-enhanced light scattering at single hot spots. *Nat. Comm.* **3**, 684 (2012)

[33] Etchegoin, P. G. & Le Ru, E. C. Basic Electromagnetic Theory of SERS in *Surface Enhanced Raman Spectroscopy: Analytical, Biophysical and Life Science Applications* (Wiley, 2011)

[34] Kleinman, S. L. et al. Structure enhancement factor relationships in single gold nanoantennas by surface-enhanced Raman excitation spectroscopy. *J. Am. Chem. Soc.* **135**, 301 – 308 (2013).

[35] C. D'Andrea et al., Red shifted spectral dependence of the SERS enhancement in a ranfdom array of gold nanoparticles covered with a silica shell: extinction versus scattering. *J. Opt.* **17**, 114016 (2015)

[36] Nakamura, K., et al. Conformational changes in seventeen cystine disulfide bridges of bovine serum albumin. *FEBS Lett.* **417**, 375-378 (1997)

[37] Lord, R. C., & Yu, N. T. Laser-excited Raman spectroscopy of biomolecules: I. Native lysozyme and its constituent amino acids. *J. Mol. Bio.* ***50***, 509-524 (1970).

[38] Dongmao, Z. et al. Gold nanoparticles can induce the formation of protein-based aggregates at physiological pH. *Nano Lett.* **9**, 666-671 (2009)
